# Short term supplementation with cranberry extract modulates gut microbiota in human and displays a bifidogenic effect

**DOI:** 10.1038/s41522-024-00493-w

**Published:** 2024-03-06

**Authors:** Jacob Lessard-Lord, Charlène Roussel, Joseph Lupien-Meilleur, Pamela Généreux, Véronique Richard, Valérie Guay, Denis Roy, Yves Desjardins

**Affiliations:** 1 Institute of Nutrition and Functional Foods (INAF), Faculty of Agriculture and Food Sciences, Laval University, Québec, QC Canada; 2 Nutrition, Health and Society Centre (NUTRISS), INAF, Laval University, Québec, QC Canada; 3 Department of Plant Science, Faculty of Agriculture and Food Sciences, Laval University, Québec, QC Canada; 4 Canada Excellence Research Chair on the Microbiome-Endocannabinoidome Axis in Metabolic Health, Laval University, Québec, QC Canada; 5 Department of Food Science, Faculty of Agriculture and Food Sciences, Laval University, Québec, QC Canada

**Keywords:** Clinical microbiology, Applied microbiology

## Abstract

Cranberry is associated with multiple health benefits, which are mostly attributed to its high content of (poly)phenols, particularly flavan-3-ols. However, clinical trials attempting to demonstrate these positive effects have yielded heterogeneous results, partly due to the high inter-individual variability associated with gut microbiota interaction with these molecules. In fact, several studies have demonstrated the ability of these molecules to modulate the gut microbiota in animal and in vitro models, but there is a scarcity of information in human subjects. In addition, it has been recently reported that cranberry also contains high concentrations of oligosaccharides, which could contribute to its bioactivity. Hence, the aim of this study was to fully characterize the (poly)phenolic and oligosaccharidic contents of a commercially available cranberry extract and evaluate its capacity to positively modulate the gut microbiota of 28 human subjects. After only four days, the (poly)phenols and oligosaccharides-rich cranberry extract, induced a strong bifidogenic effect, along with an increase in the abundance of several butyrate-producing bacteria, such as *Clostridium* and *Anaerobutyricum*. Plasmatic and fecal short-chain fatty acids profiles were also altered by the cranberry extract with a decrease in acetate ratio and an increase in butyrate ratio. Finally, to characterize the inter-individual variability, we stratified the participants according to the alterations observed in the fecal microbiota following supplementation. Interestingly, individuals having a microbiota characterized by the presence of *Prevotella* benefited from an increase in *Faecalibacterium* with the cranberry extract supplementation.

## Introduction

Consumption of cranberry (*Vaccinium macrocarpon*) is associated with multiple health benefits, notably reducing the incidence of urinary tract infections and preventing cardiovascular and neurodegenerative diseases^1–5^. These positive effects have been primarily attributed to their high concentrations of (poly)phenols^4,6,7^. Cranberry is rich in various (poly)phenolic compounds, including phenolic acids, anthocyanins and flavonols, with the most prevalent being flavan-3-ols^7,8^. Notably, cranberry is one of the few dietary sources that contains a specific type of oligomeric flavan-3-ols, called A-type proanthocyanidins^7,8^.

The positive effects of (poly)phenols on health were believed to stem from the high antioxidant activity in the host. However, research has shown that these molecules are poorly absorbed (< 10%) in the small intestine and a significant portion (> 90%) reaches the colon^9–11^. Hence, it is believed that polyphenols exert their health effects through their action on the gut microbiota in the colon^12,13^. Indeed, (poly)phenols interact bidirectionally within the gut microbiota. They directly alter its composition by inhibiting the growth of pathogenic bacteria (antimicrobial effect) and stimulating the growth of beneficial ones (prebiotic-like effect). This dual mode of action led to the introduction of the concept of “*duplibiotics*” by our research group^14^. Conversely, the gut microbiota can break down (poly)phenols into bioavailable and potentially bioactive metabolites^11,12^.

Clinical trials investigating the health effects of cranberries have yielded mixed and varied results, most likely due to the large inter-individual variability in the capacity of the gut microbiota to convert cranberry (poly)phenols into bioavailable and potentially bioactive metabolites^15–18^. Previous studies have attempted to classify individuals into distinct groups based on their metabolic profiles following the ingestion of cranberry flavan-3-ols. These classifications were based on the differential production of specific metabolites, such as 5-(3′,4′-dihydroxyphenyl)-γ-valerolactone, and were not linked to the different gut enterotypes. This approach sought to gain insights into the underlying factors contributing to the inter-individual variability observed in the health effects of cranberry^18^. However, it was recently demonstrated that the most abundant (poly)phenols family in cranberry, flavan-3-ols oligomers, are not metabolized by the gut microbiota^19–21^. Therefore, since host microorganisms are unable to directly utilize flavan-3-ols oligomers, they do not strictly correspond to the definition of prebiotic according to Gibson et al. ^22^. However, these molecules exhibit a strong prebiotic-like effect, that is they have the ability to induce beneficial effects through the modulation of the microbiota^14^. Most notably, cranberry (poly)phenols can enhance the growth and abundance of *Akkermansia muciniphila*, a mucosal bacterial species associated with numerous health benefits, including potential anti-obesity effect^23–27^. As the different metabolic profiles alone cannot explain the inter-individual variability linked to the health benefits derived from cranberry consumption, it is imperative to explore other potential sources of variability. One should not neglect the impact of cranberry consumption on the modulation of gut microbiota composition to explain inter-individual variation.

Coleman and Ferreira introduced a paradigm shift regarding the constituents responsible for the bioactivity of cranberry^28^. Their research suggests that complex carbohydrates, such as arabinoxyloglucan and pectic oligosaccharides, present in high concentrations, may play a significant role in mediating the beneficial effects of cranberry. This highlights the importance of considering these specific oligosaccharides as potential contributors to cranberry’s bioactivity and their impact on health outcomes^28^. In fact, commercially available flavan-3-ols-rich cranberry extracts contain approximately 15% (*w/*w) of flavan-3-ols, whereas oligosaccharides themselves represent about 20% of the total extract mass^28^. This suggests that the oligosaccharides present in cranberry extracts may play a significant role in cranberry’s health benefits, through a prebiotic effect^28,29^.

Thus, the aim of this study was to evaluate the effect of a 4-day supplementation with a purified cranberry extract (Prebiocran^TM^), containing both (poly)phenols and oligosaccharides, on the composition and function of the fecal microbiota in a human clinical trial involving 39 healthy individuals. Fecal microbiota composition was analyzed by 16 S rRNA sequencing and short-chain fatty acids (SCFA) were quantified in both feces and plasma. Moreover, as part of the study, participants were categorized into enterotypes based on the cranberry extract-induced changes in the abundance of bacterial genera within their gut microbiota. This stratification unravelled specific responses depending on the initial gut microbiota composition and clarified the relationship between cranberry extract supplementation and inter-individual variability.

## Results

### Cranberry extract contains high quantities of oligosaccharides and (poly)phenols

**Fig. 1:**
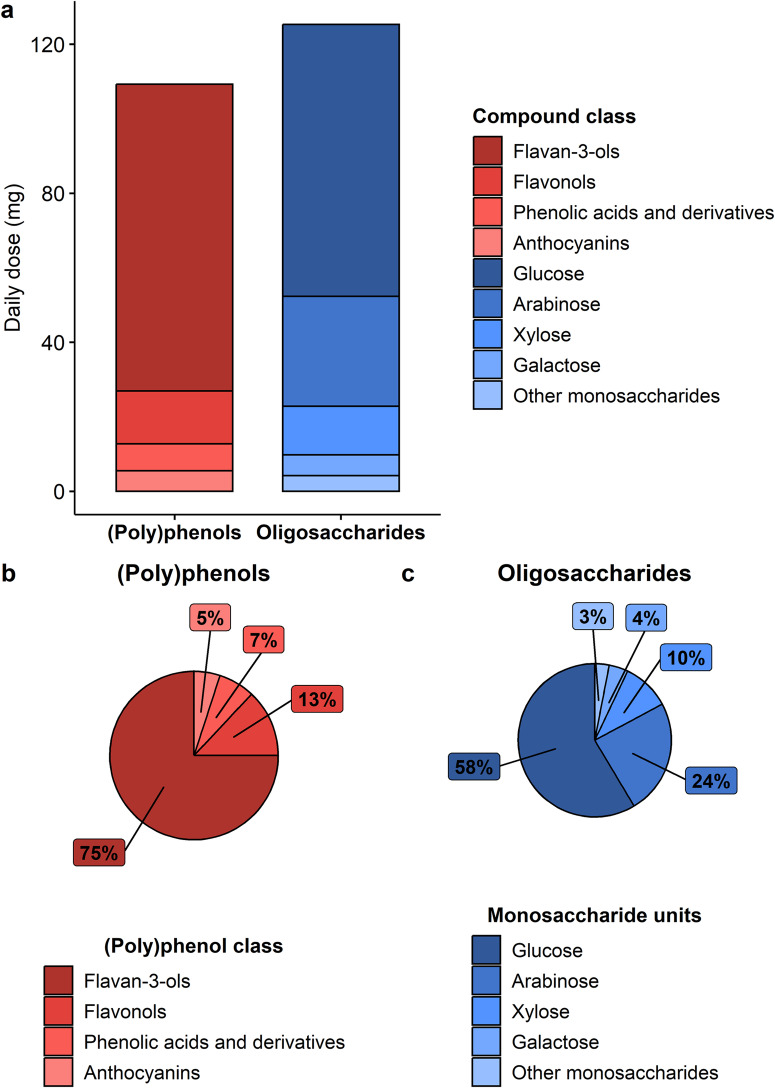
Characterization of the cranberry extract. **a** Daily dose of (poly)phenols and oligosaccharides provided by the cranberry extract. Proportion of each (poly)phenols class of the total (poly)phenolic content (**b**) and of each monosaccharide of the total complex carbohydrates content (**c**).

The cranberry extract supplement used in this study provided 109 mg of (poly)phenols and 125 mg of oligosaccharides per day to the participants (Table 1 and Fig. 1a). Flavan-3-ols (82.3 mg/day) accounted for 75% of the total (poly)phenolic content. However, the extract also contained additional (poly)phenols such as flavonols, phenolic acids and anthocyanins (Fig. 1b). A detailed characterization of the (poly)phenols provided by the cranberry extract is presented in Supplementary Table 1. Monosaccharide units forming the cranberry oligosaccharides were determined after acid hydrolysis (to release monosaccharide units from the oligosaccharides) and consisted mainly of glucose (58%), arabinose (24%), xylose (10%), and galactose (4%) (Fig. 1c). Monomeric units of pectic oligosaccharides, namely galacturonic acid, represented less than 1% of the total oligosaccharides content.

**Table 1:** Characterization of the (poly)phenols and complex carbohydrates in the cranberry extract

Compound	Daily dose (mg)
Flavan-3-ols^a^	82.3 ± 0.2
Flavonols	14.18 ± 0.12
Phenolic acids and derivatives	7.25 ± 0.05
Anthocyanins	5.50 ± 0.13
**Total (poly)phenols**	**109.3** ± **0.5**
Glucose	73 ± 5
Arabinose	29 ± 4
Xylose	13.0 ± 0.3
Galactose	5.6 ± 0.7
Fructose	1.7 ± 0.4
Rhamnose	1.30 ± 0.19
Galacturonic acid	0.9 ± 0.3
Fucose	0.37 ± 0.09
**Total complex carbohydrates**^**b**^	**125** ± **9**

^a^Determined by phloroglucinolysis.

^b^Monosaccharides composition was analyzed following acid hydrolysis of complex carbohydrates. Mean and standard deviation were calculated based on the results of triplicates.

### Short-term consumption of cranberry extract strongly modulates the fecal microbiota within four days

39 healthy subjects were included in the trial (Table 2); plasma samples were collected from all of them, while 28 provided fecal samples before (V1) and after (V2) the cranberry extract supplementation. Composition of the fecal microbiota was analyzed by 16S rRNA sequencing and is presented in Supplementary Figs. 1 to 4.

**Table 2:** Age, gender and anthropometric measurements of the participants enrolled in this study

Characteristics	Value	
	Mean	SD
Age (years)	36	11
Sex		
Female	24	
Male	15	
Weight (kg)	67	13
BMI (kg/m^2^)	23	4
Waist circumference (cm)	82	13

**Fig. 2:**
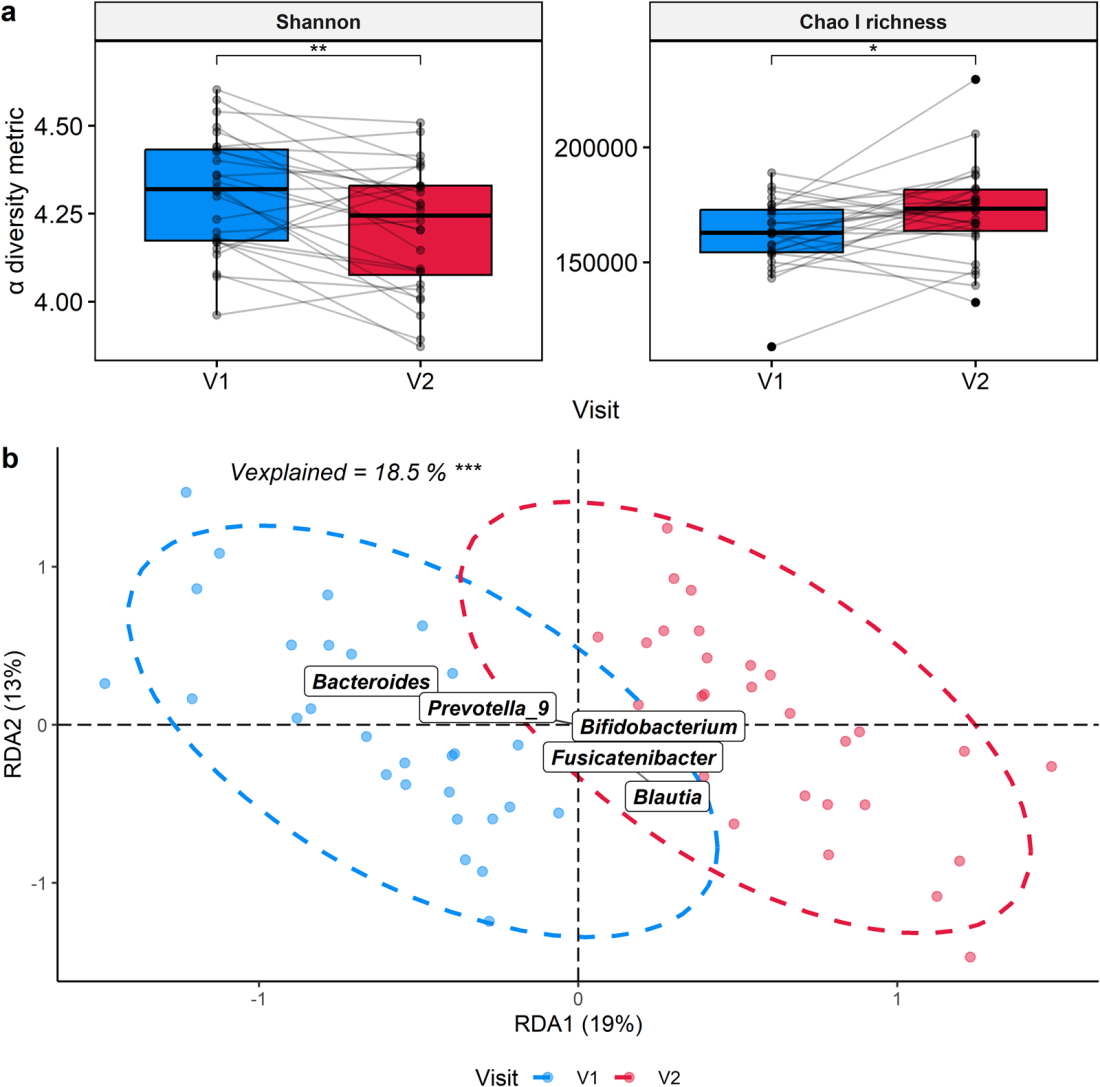
Global effect of the cranberry extract supplementation on the diversity and richness of the fecal microbiota. **a** The Shannon and Chao1 indexes were measured before (V1) and after (V2) cranberry extract supplementation. Each sample is represented by a point and a line connects paired samples from the same subjects between V1 and V2. Statistical significance was assessed with paired t-test adjusted for multiple comparisons using Benjamini & Hochberg method. The center of the boxplots represents the median, the borders represent the first and third quartiles and the whiskers represent the minimum and maximum. **b** Bacteria genus-based db-RDA discriminating samples collected before (V1) and after (V2) cranberry extract supplementation. Each sample is represented by a point. Statistical significance was assessed with a permutational multivariate analysis of variance (PERMANOVA). Results of the statistical analysis were represented with asterisks (*p ≤ 0.05, ** *p* ≤ 0.01, ***p ≤ 0.001, ****p ≤ 0.0001). % of the total variability explained by each axis is reported in axis titles.

Initially, the impact of a four-day supplementation with the cranberry extract on the fecal microbiota of all participants was assessed. The α-diversity was measured by Shannon index and richness by Chao1 index, while β-diversity was calculated by distance-based redundancy analysis (db-RDA) (Fig. 2). The administration of cranberry extract for four days resulted in a significant increase in species richness (*p* ≤ 0.05) and a decrease in α-diversity (*p* ≤ 0.01), indicating reduced evenness among bacterial species, as shown in Fig. 2a. Given the substantial variability in bacterial community composition at the species level among participants, the analysis initially focused on examining general trends across participants at the genus level. This approach allowed for a broader understanding of the patterns and associations within bacterial communities that were more consistent across the study population. Consumption of cranberry extract had a significant impact on microbial β-diversity, as shown in Fig. 2b. The first component of the analysis effectively distinguished samples collected before (V1) and after (V2) cranberry supplementation, explaining 18.5% of the total variability in the microbiota (*p* ≤ 0.001). This finding suggests that cranberry supplementation influenced the overall composition and structure of the gut microbiota. In the V1 samples, the genera *Bacteroides* and *Prevotella*_*9* were prominent, while in the V2 samples, the genera *Bifidobacterium*, *Fusicatenibacter*, and *Blautia* exhibited stronger associations (Fig. 2b and Supplementary Fig. 5).

### Bifidobacterium is increased with the cranberry extract at the expense of Bacteroides

**Fig. 3:**
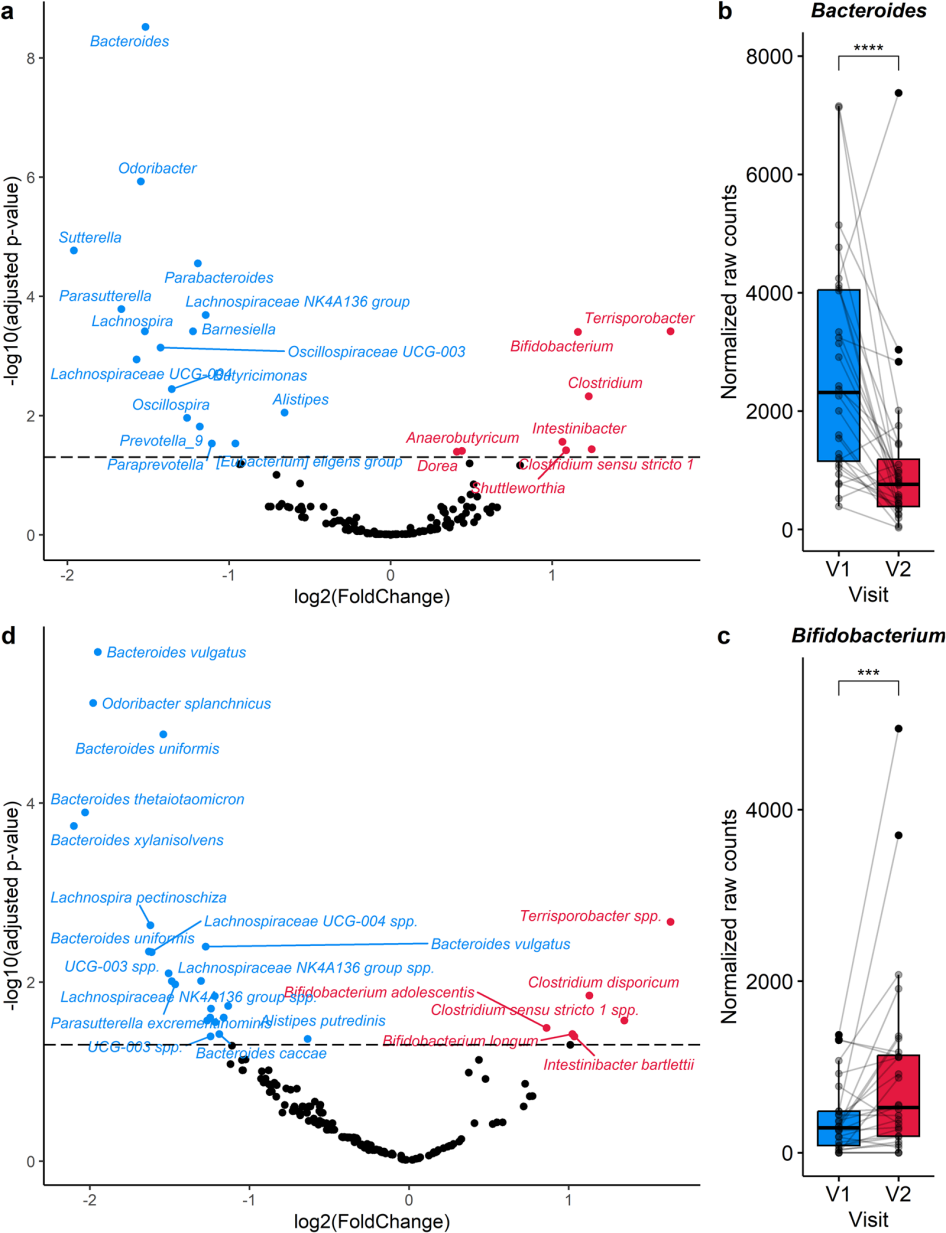
Impact of cranberry extract supplementation on the abundance of bacterial genera and species using DESeq2 analysis. Volcano plots highlighting significant genera (**a**) and species (**d**) that are modulated by the cranberry extract supplementation. Adjusted *p*-values were obtained with Wald test adjusted for multiple comparisons using the Benjamini & Hochberg method. Dotted line at adjusted *p* = 0.05 indicates the statistical significance. **b**, **c** Boxplots showing the paired microbiota samples for the selected genera *Bacteroides* and *Bifidobacterium*. Statistical significance, as determined on the volcano plot, was represented with asterisks (*p ≤ 0.05, **p ≤ 0.01, ***p ≤ 0.001, ****p ≤ 0.0001). The center of the boxplots represents the median, the borders represent the first and third quartiles and the whiskers represent the minimum and maximum.

To further determine whether the abundance of the microbial genera was affected by the cranberry supplementation, a DESeq analysis was performed (Fig. 3a and Supplementary Table 2). Among the most striking results, the abundance of *Bacteroides* was significantly reduced by the cranberry extract (*p* ≤ 0.0001, Fig. 3b), while that of *Bifidobacterium* was significantly increased (*p* ≤ 0.001, Fig. 3c), as observed with the db-RDA. The treatment led to a decrease of the abundance of certain genera, including *Parabacteroides* (*p* ≤ 0.0001), *Prevotella_9* (*p* ≤ 0.05), and *Paraprevotella* (*p* ≤ 0.05). On the other hand, the abundance of the genera *Terrisporobacter* (*p* ≤ 0.001), *Clostridium* (*p* ≤ 0.001), *Clostridium sensu stricto 1* (*p* ≤ 0.05), *Anaerobutyricum* (*p* ≤ 0.05), and *Dorea* (*p* ≤ 0.05) was increased by the cranberry supplementation (Fig. 3a and Supplementary Fig. 6). To gain further insights into the effects of the cranberry extract on the microbiota, DESeq analysis was conducted focusing on species belonging to the significantly modulated genera (Fig. 3d and Supplementary Table 3). For *Bacteroides*, five species were specifically decreased by the cranberry supplementation, namely *Bacteroides caccae* (*p* ≤ 0.05)*, Bacteroides thetaiotaomicron* (*p* ≤ 0.001), *Bacteroides uniformis* (*p* ≤ 0.01), *Bacteroides vulgatus* (*p* ≤ 0.01) and *Bacteroides xylanisolvens* (*p* ≤ 0.001) (Fig. 3d and Supplementary Fig. 7a). For *Bifidobacterium*, two species were increased with the cranberry supplementation, namely *Bifidobacterium adolescentis* (*p* ≤ 0.05) and *Bifidobacterium longum* (*p* ≤ 0.05), while there was no significant change (*p* > 0.05) for *Bifidobacterium animalis*, *Bifidobacterium bifidum* and *Bifidobacterium catenulatum* (Fig. 3d and Supplementary Fig. 7b).

**Fig. 4:**
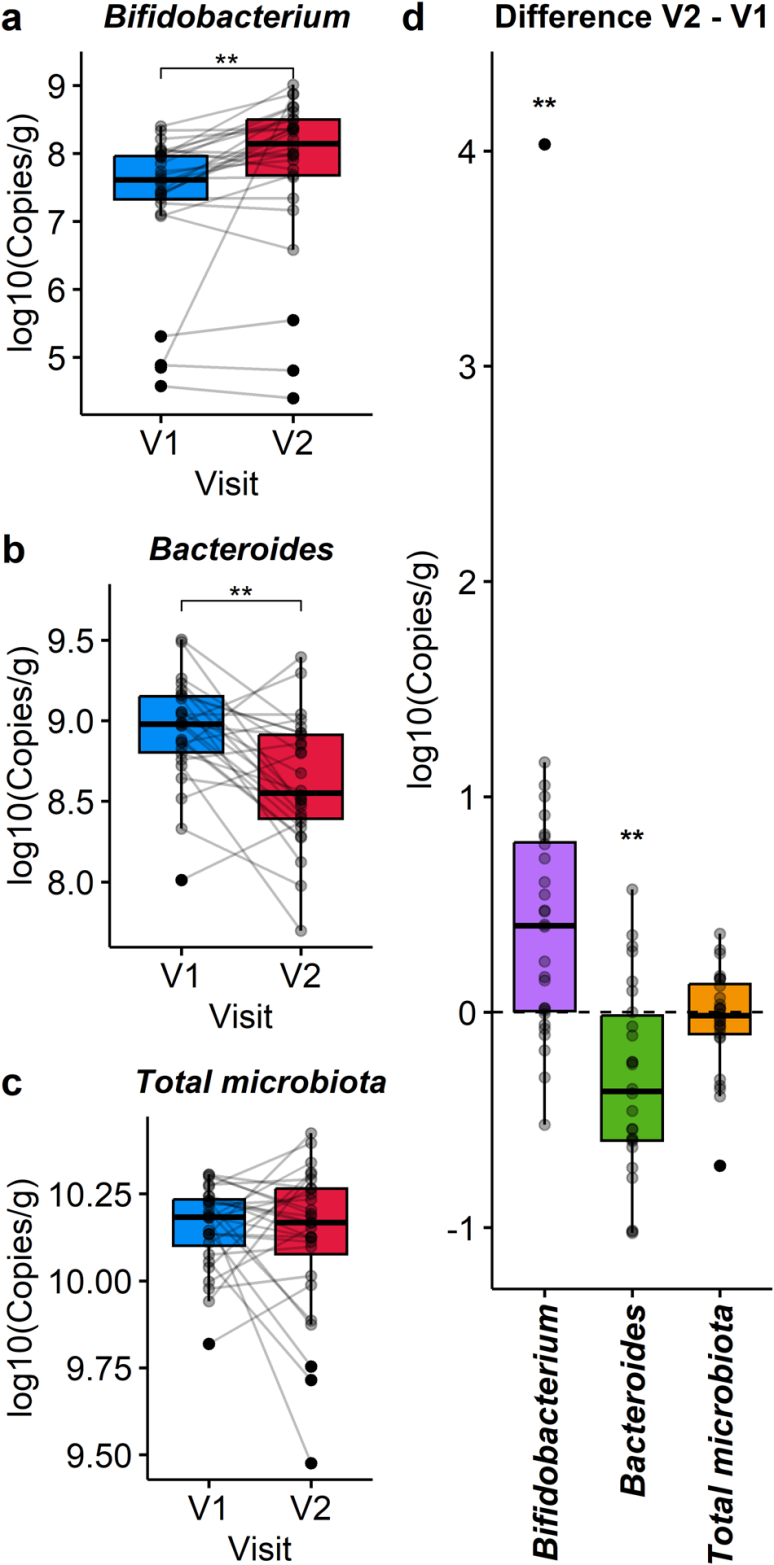
Validation of the main effects induced by the cranberry extract supplementation by quantitative PCR. Results are represented as boxplots showing the paired microbiota samples (**a**–**c**) and the difference between V2 – V1 (**d**) of the selected genera *Bifidobacterium* and *Bacteroides*, as well as the total microbiota. Statistical significance was assessed with a paired Wilcoxon test adjusted for multiple comparisons using the Benjamini & Hochberg method. Results of the statistical analysis were represented with asterisks (*p ≤ 0.05, **p ≤ 0.01, ***p ≤ 0.001, ****p ≤ 0.0001). The center of the boxplots represents the median, the borders represent the first and third quartiles and the whiskers represent the minimum and maximum.

Quantitative PCR analysis was conducted to validate that cranberry extract supplementation effectively modulated the absolute abundance of *Bifidobacterium* and *Bacteroides* in fecal samples. We confirmed that the number of *Bifidobacterium* was significantly stimulated by the supplementation (*p* ≤ 0.01, Fig. 4a, d), while Bacteroides was significantly decreased (*p* ≤ 0.01, Fig. 4b, d). However, the supplementation did not significantly impact the total bacteria concentration in the fecal samples (Fig. 4c, d).

**Fig. 5:**
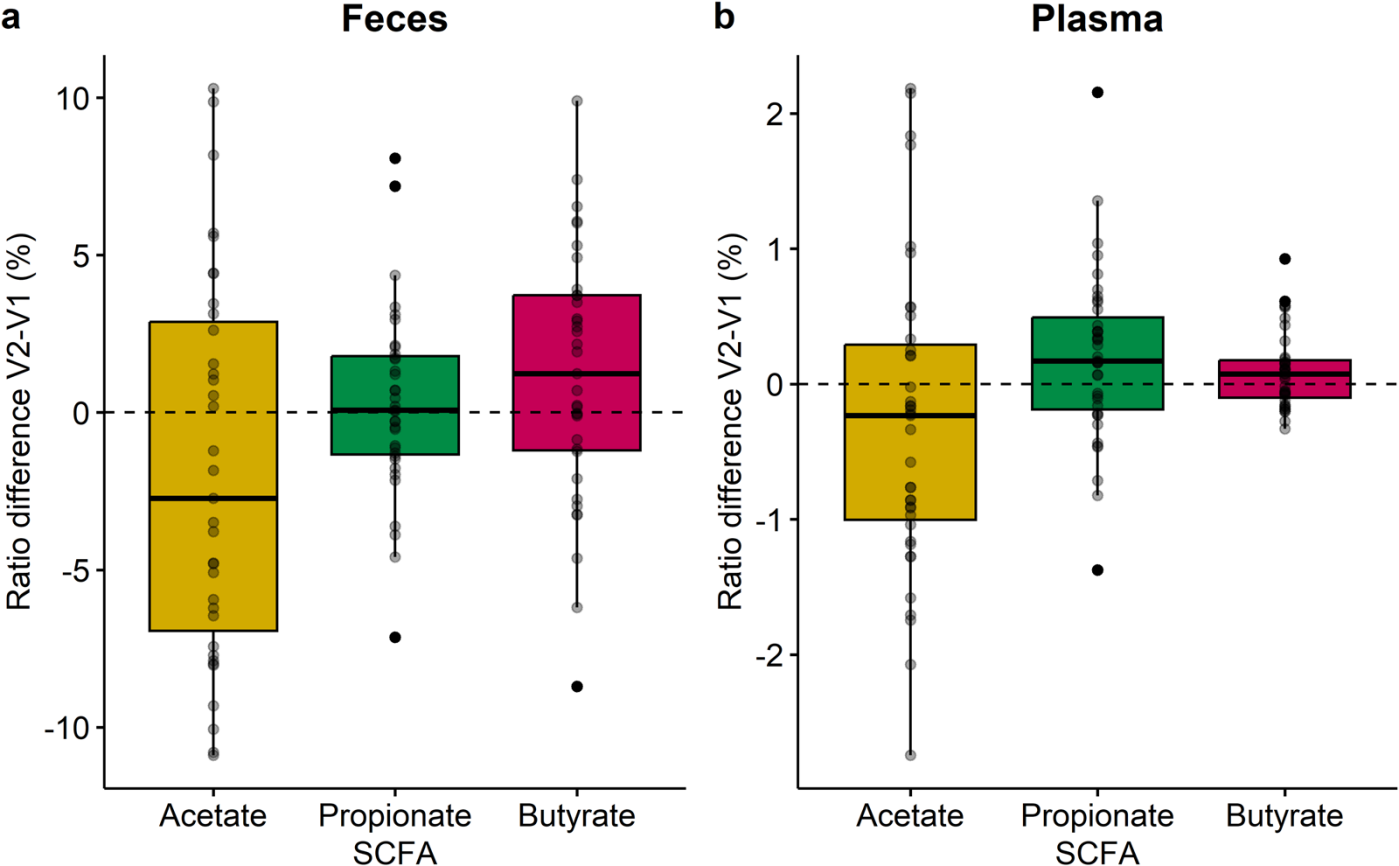
Ratio disparity of the three major SCFA after cranberry extract supplementation. Results in feces (*n* = 28, **a**) and in plasma (*n* = 39, **b**) are represented as boxplots. The ratio of each major SCFA (acetate, propionate and butyrate) was calculated by dividing its concentration by the sum of the major SCFA and expressed as percentage. The difference between V2 – V1 was visualized to demonstrate the effect of the cranberry extract supplementation on the different ratios. Each subject is represented as a point on the plot. A dotted line was added at the ratio difference V2 – V1 (%) = 0 to indicate no change between V1 and V2. Statistical significance was assessed with paired Wilcoxon test adjusted for multiple comparisons using the Benjamini & Hochberg method. No statistical difference was found between samples collected at V1 and V2. Center of the boxplots represents the median, the borders represent the first and third quartiles and the whiskers represent the minimum and maximum.

To investigate the effect of the cranberry extract supplementation on the function of the gut microbiota, SCFA were quantified in feces (Fig. 5a) and plasma (Fig. 5b) samples. Although no significant differences were observed for the ratio of the three major SCFA, due to high inter-individual variability, interesting trends could still be observed. In both matrices, acetate production was globally decreased (*p* = 0.1 in feces and *p* = 0.09 in plasma), while butyrate was increased by the cranberry intake (*p* = 0.1 in feces and *p* = 0.09 in plasma) (Fig. 5). In addition, the rise of propionate was only observed in plasma samples following the supplementation with cranberry extract (*p* = 0.09, Fig. 5b).

### Faecalibacterium bloomed depending on the initial microbial composition of the participants following cranberry extract supplementation

**Fig. 6:**
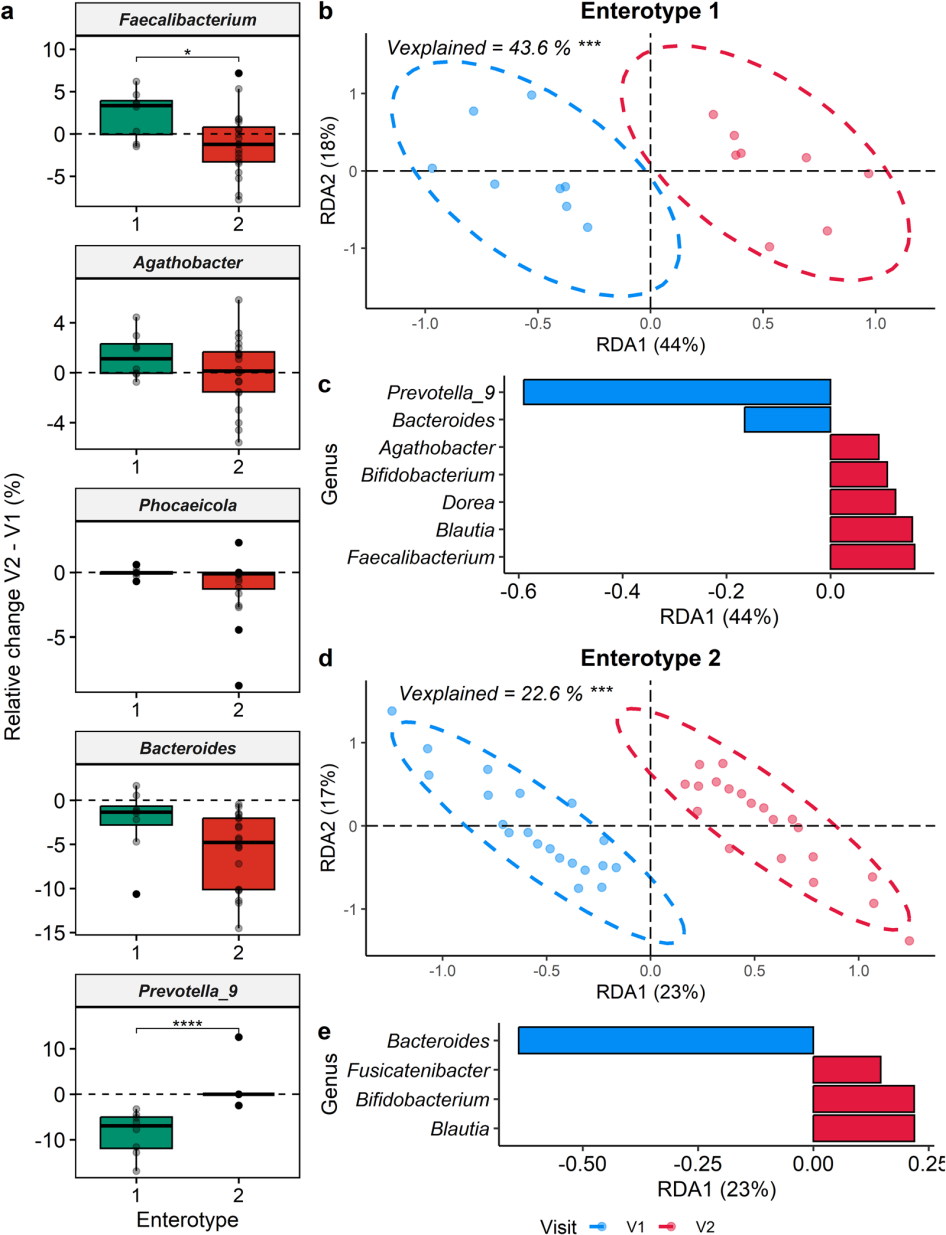
Impact of the cranberry extract supplementation on fecal microbiota depending on the enterotypes. **a** Boxplots of the main bacterial genera discriminating the two enterotypes are represented. Statistical significance was assessed with Wilcoxon test adjusted for multiple comparisons using the Benjamini & Hochberg method. Center of the boxplots represents the median, the borders represent the first and third quartiles and the whiskers represent the minimum and maximum. db-RDA performed with subjects from enterotype 1 (**b**, **c**) and enterotype 2 (**d**, **e**). Each sample is represented by a point. % of the total variability explained by each axis is reported between parenthesis in axis titles. Statistical significance was assessed with a permutational multivariate analysis of variance (PERMANOVA). Results of the statistical analysis were represented with asterisks (* *p* ≤ 0.05, ** *p* ≤ 0.01, *** *p* ≤ 0.001, **** *p* ≤ 0.0001).

Furthermore, we explored the microbiota inter-individual variability associated with cranberry supplementation. The participants enrolled in this study were clustered into two enterotypes (Supplementary Fig. 8), based on the principal component analysis (PCA) using the difference in the relative abundance of genera detected in their fecal microbiota (relative abundance of genus X at V2 – relative abundance of genus X at V1). The two enterotypes were obtained following k-means clustering based on the PCA. The first principal component (PC1) separated the two clusters, explaining over 21% of the total variation on the score plot (Supplementary Fig. 8a). As represented on the loading plot (Supplementary Fig. 8b), the partition of the two enterotypes was mainly guided by the modulation of *Faecalibacterium* and *Prevotella_9*, and to a lesser extent by other genera, such as *Agathobacter* (genus comprising the important former *Eubacterium rectale*), *Phocaeicola* and *Bacteroides*. Following the cranberry supplementation, enterotype 1 was characterized by an increase of the abundance of *Faecalibacterium* (*p* ≤ 0.05) and *Agathobacter* (*p* = 0.3) and a reduction of *Prevotella_9* (*p* ≤ 0.0001), while enterotype 2 was characterized by a greater reduction in the abundance of *Phocaeicola* (*p* = .2) and *Bacteroides* (*p* = 0.2) (Fig. 6a and Supplementary Fig. 9). Among the 28 participants included in the study, eight individuals were classified as belonging to enterotype 1, while the remaining 20 individuals were assigned to enterotype 2.

However, no significant difference between the two enterotypes (*p* > .05) was observed for the effect of the cranberry extract on Shannon α-diversity, and Chao1 richness indexes (Supplementary Fig. 10). The effect of the treatment on β-diversity was also assessed by performing a db-RDA on each enterotype separately (Supplementary Fig. 6b–e). This parameter was significantly affected (*p* ≤ .001) for both enterotypes, with the first component separating samples from V1 and V2 and explaining 43.6% for enterotype 1 and 22.6% for enterotype 2 of the total microbiota variation. For both enterotypes, *Bacteroides* was associated with V1, while *Bifidobacterium* and *Blautia* were linked with V2 (Fig. 6b–e). Remarkably, the bifidogenic effect induced by cranberry consumption was preserved in both enterotypes. However, for enterotype 1, *Prevotella_9* was also related to V1 and *Faecalibacterium*, *Dorea* and *Agathobacter* were associated with V2 (Fig. 6b–e).

**Fig. 7:**
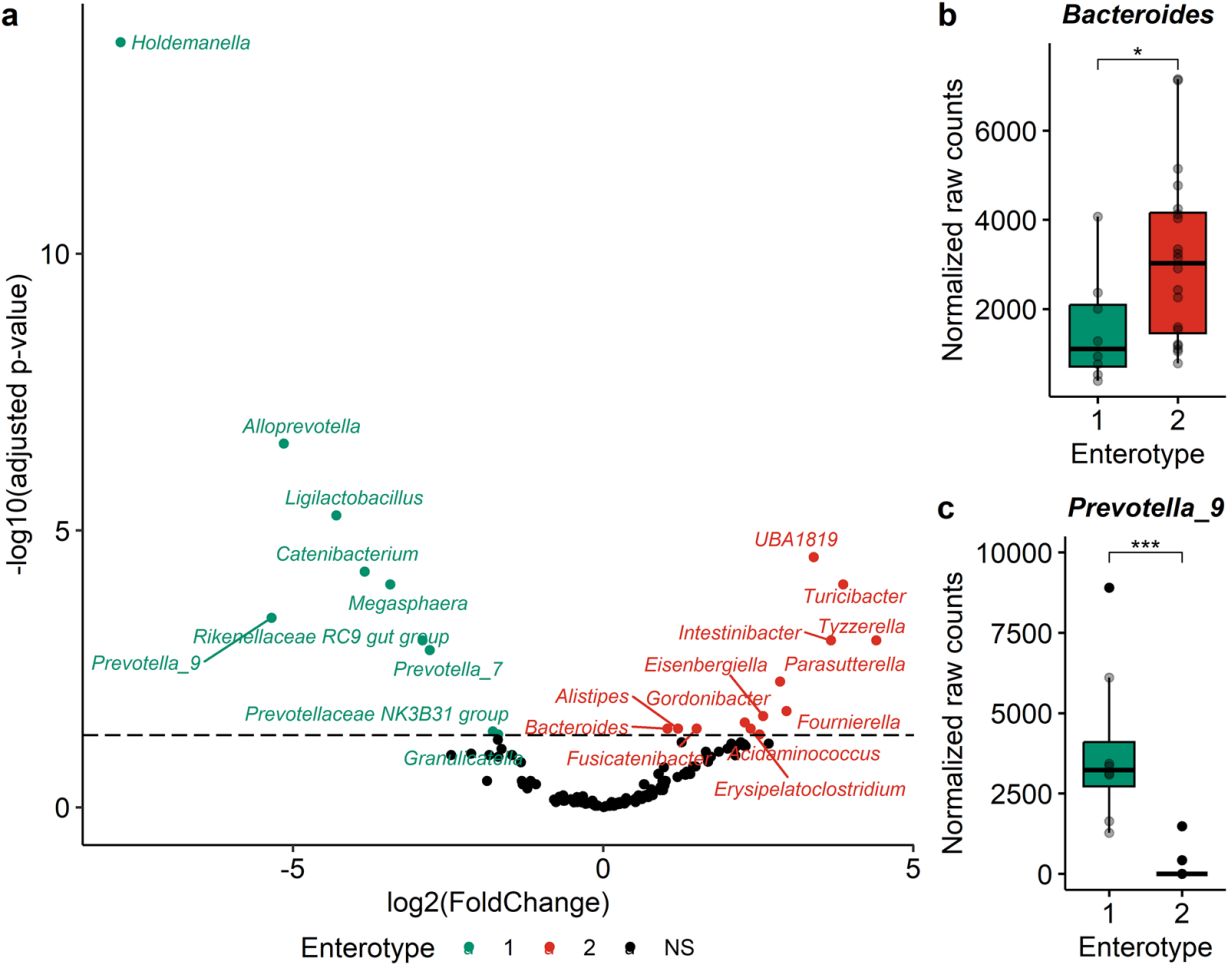
Characterization of the microbial dissimilarity abundance between enterotypes, prior to cranberry extract supplementation using DESeq2 analysis. **a** Volcano plot highlighting significant genera (adjusted *p* > 0.05) that are different between enterotypes prior to the cranberry extract supplementation. Adjusted *p*-values were obtained with Wald test adjusted for multiple comparisons using the Benjamini & Hochberg method. Dotted line at adjusted *p* = .05 indicates the statistical significance. **b**, **c** Boxplots showing the paired microbiota samples for the selected genera *Bacteroides* and *Prevotella_9*. Statistical significance, as determined on the volcano plot, was represented with asterisks (*p ≤ 0.05, **p ≤ 0.01, ***p ≤ 0.001, ****p ≤ 0.0001). Center of the boxplots represents the median, the borders represent the first and third quartiles and the whiskers represent the minimum and maximum.

In order to understand the origin of these differences in the modulation of the fecal microbiota by the cranberry extract supplementation, DESeq analysis was performed with samples collected before the supplementation to assess the initial difference between the enterotypes (Fig. 7a and Supplementary Table 4). Interestingly, *Bacteroides* was significantly more abundant in subjects belonging to enterotype 2 (Fig. 7b, *p* ≤ 0.05), while *Prevotella_9* was almost exclusively present in samples associated to the enterotype 1 (Fig. 7c, *p* ≤ 0.001). In fact, only two participants out of twenty within enterotype 2 had a low abundance of this genus (Fig. 7c). Other genera discriminated the initial fecal microbiota (V1) of participants from enterotype 1 from those from enterotype 2 (Supplementary Fig. 11).

**Fig. 8:**
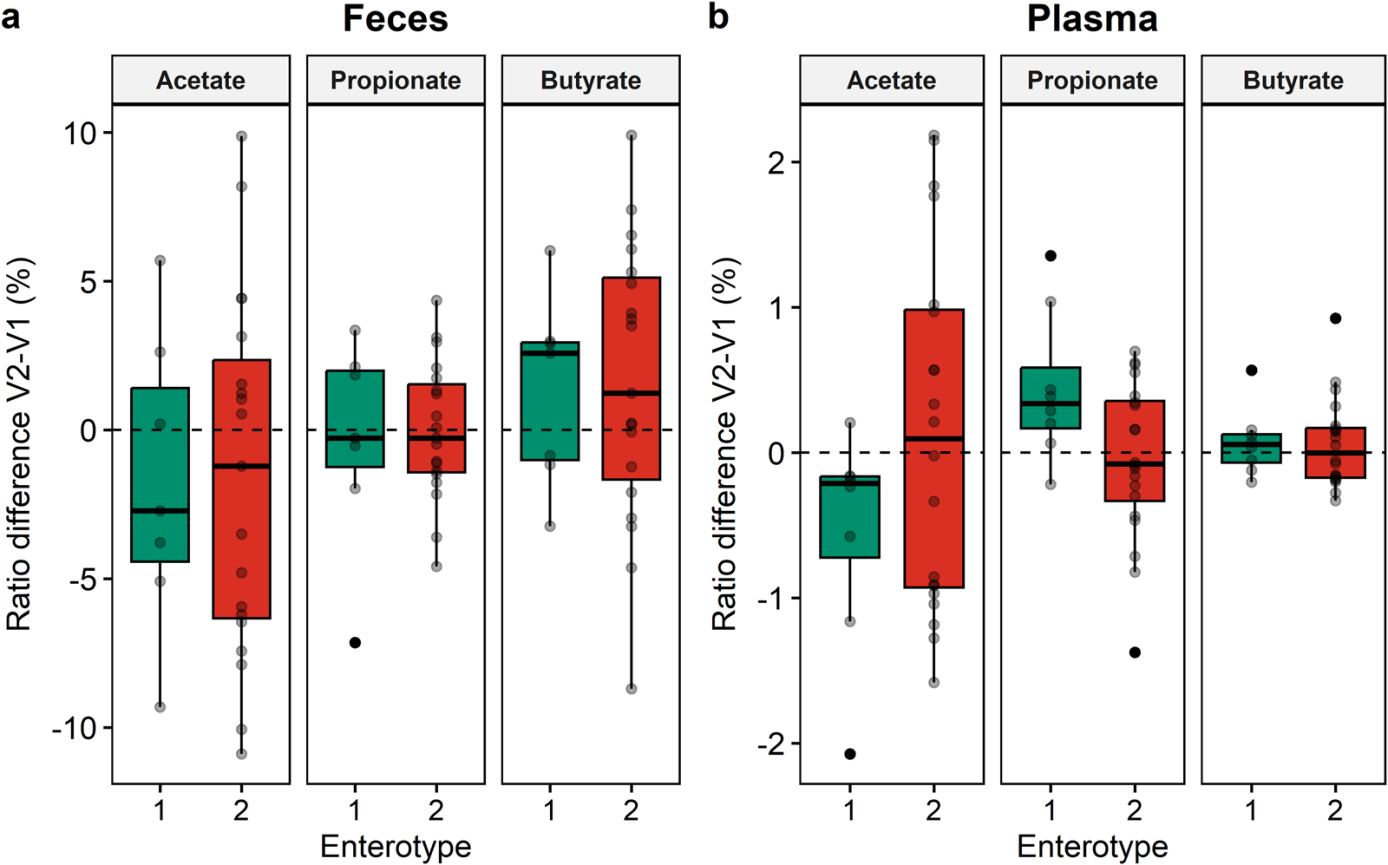
Ratio disparity of the three major SCFA between enterotypes after cranberry extract supplementation. Results in feces (**a**) and in plasma (**b**) are represented as boxplots. The ratio of each major SCFA (acetate, propionate and buryrate) was calculated, as in Fig. 5, by dividing its concentration by the sum of the major SCFA and expressed as percentage. The difference between V2 – V1 was visualized to demonstrate the effect of the cranberry extract supplementation on the different ratios for each enterotype. Each subject is represented as a point on the plot. A dotted line was added at the ratio difference V2 – V1 (%) = 0 to indicate no change between V1 and V2. Statistical significance was assessed with a paired Wilcoxon test adjusted for multiple comparisons using the Benjamini & Hochberg method. No statistical difference was found between enterotypes, nor between samples collected at V1 and V2 for each enterotype. Center of the boxplots represents the median, the borders represent the first and third quartiles and the whiskers represent the minimum and maximum.

Finally, we investigated whether there were enterotype-based differences in the production of SCFA in feces (Fig. 8a) and plasma (Fig. 8b). Comparable ratio differences (V2 – V1) for the three major SCFA were observed in fecal samples across both enterotypes, characterized by a consistent decrease in acetate levels and an increase in butyrate concentrations (Fig. 8a). Nonetheless, the cranberry extract appeared to have varying effects on plasmatic SCFA ratios between the two enterotypes, although these differences did not reach statistical significance. (Fig. 8b, *p* > 0.05). In enterotype 1 individuals, the cranberry extract treatment resulted in a global decrease in acetate levels and an increase in the proportion of propionate. Conversely, within enterotype 2 individuals, the opposite trend was observed, with a slight increase in acetate and a mild decrease in propionate proportions due to the treatment, but high inter-individual variability was observed. In addition, the ratio of butyrate was slightly increased by the cranberry extract supplementation for enterotype 1, while there was no clear modulation for enterotype 2.

## Discussion

Health effects of cranberry have historically been attributed to the antioxidant activity of the (poly)phenols in the host^9^. Since these molecules are poorly absorbed in the small intestine, the focus has shifted to their interaction with the gut microbiota^12,13^. It was initially believed that cranberry (poly)phenols, especially flavan-3-ols, could be metabolized by specific gut microbiota members into smaller bioavailable and potentially bioactive metabolites, such as phenyl-γ-valerolactones. However, cranberry flavan-3-ols are mainly A-type oligomeric proanthocyanidins, which are poorly degraded in the gut^19,21^. Therefore, the purpose of the present study was to fully characterize a commercially available cranberry extract (Prebiocran^TM^) to determine the nature and the amount of (poly)phenols and oligosaccharides contained in the extract and to assess their capacity to positively modulate the gut microbiota of 28 healthy subjects within a 4-day supplementation.

Cranberry extract contains a complex mixture of (poly)phenols and oligosaccharides, but few studies assessed the oligosaccharides content of cranberry^29–35^. Interestingly, oligosaccharides were more abundant than (poly)phenols in the purified cranberry extract used in our study. Monosaccharide composition following acid hydrolysis of oligosaccharides in the cranberry extract is coherent with that reported by Sun et al. in purified oligosaccharides fraction from pectinase-treated cranberry pomace^30^. Oligosaccharides in this fraction were mostly composed of glucose (47%), arabinose (25%), xylose (23%) and galactose (5%)^30^, while the oligosaccharides in the cranberry extract used in our study were mainly formed of the same monosaccharides, but with the following proportions: 58%, 24%, 10% and 4%. Hence, we confirmed that the majority of the oligosaccharides in the cranberry extract were arabinoxyloglucans. Only a small amount of galacturonic acid was detected in the cranberry extract (< 1% of the total oligosaccharide content), indicating that pectic oligosaccharides were only present in small amounts. As previously reported for cranberry, flavan-3-ols, particularly A-type proanthocyanidins, were the most abundant (poly)phenol class in the extract^7,8^. The combined action of (poly)phenols and oligosaccharides likely contributes to the overall impact of cranberry extract on the gut microbiota.

It is worth noting that hypromellose (hydroxypropyl methyl cellulose), the filling agent used in the capsules, could also have an impact on the gut microbiota. Naimi et al. reported that hypromellose decreased bacterial density and α-diversity in an in vitro model^36^. However, we did not observe that effect in our study.

To our knowledge, this is the first study to demonstrate the effect of a short-term supplementation with cranberry extract, containing both (poly)phenols and oligosaccharides, on the fecal microbiota of human subjects. Interestingly, the consumption of the cranberry extract successfully modulated the fecal microbiota of the participants included in this study with a strong bifidogenic effect. This effect is commonly associated with supplementation of prebiotic fibers, such as inulin and fructooligosaccharides, as first reported by Gibson & Roberfroid^37^ and confirmed by many other studies^38–42^. In the present study, *Bifidobacterium* was significantly increased with the cranberry extract providing low amounts of (poly)phenols (109.3 mg/day) and oligosaccharides (125 mg/day, mainly arabinoxyloglucan). The bifidogenic effect was concomitant to a decrease in *Bacteroides* abundance, which is recognized to efficiently metabolize complex carbohydrates, such as xylans and arabinoxylans, among others^43,44^. We surmise that cranberry (poly)phenols have an antimicrobial effect on *Bacteroides*, allowing *Bifidobacterium* to consume cranberry oligosaccharides and occupy its microbial niche (prebiotic effect). In fact, the two species of *Bifidobacterium* that were significantly increased by the cranberry extract, namely *B. adolescentis* and *B. longum*, are known to be great degrader of xylo- and arabinoxylan-oligosaccharides^45,46^. Hence, the combination of (poly)phenols and oligosaccharides in the cranberry extract is coherent with our concept of “duplibiotic”, which is a unabsorbed substrate modulating the gut by both antimicrobial and prebiotic effects^14^.

Apart from a direct antibacterial effect, the relative reduction of *Bacteroides* population might result from the symbiotic relationship this genus established with *Bifidobacterium* in the degradation of cranberry carbohydrates. As it has been demonstrated for arabinogalactan^47^, this cooperation could lead to an increase in *Bifidobacterium* population and to the acidification of the medium due to the important concentrations of formate, acetate and lactate produced by this genus when fermenting cranberry xyloglucans^48^ that could then be detrimental to *Bacteroides*. Formate, acetate and lactate exert antibacterial effects and pH reduction to which *Bacteroides* is sensitive^49^.

Although cranberry extract supplementation resulted in a bifidogenic effect in most subjects, a portion of the subjects did not exhibit the same response to the supplementation. This inherent variability in individuals is a common and frequently observed phenomenon in many supplementation studies. A similar pattern was previously noted in a 4-week study involving daily supplementation of 10 g of oligofructose-enriched inulin^50^.

The knowledge regarding the effects of cranberry on the gut microbiota in human subjects is relatively limited compared to the abundance of studies conducted in mice and in vitro settings. For instance, a bifidogenic effect was observed following the consumption of cranberry juice (providing 161 mg of (poly)phenols and an unreported amount of oligosaccharides) for 15 days in 10 postmenopausal women^51^. Along with *Bifidobacterium*, cranberry juice stimulated the growth of *Prevotella*, *Clostridium* XIVa and *Eggerthella*, while *Bacteroides* was not affected by the cranberry juice intake. Interestingly, *Clostridium* XIVa, a known cluster of butyrate-producers, was increased and other studies also reported the stimulation of genera associated with the production of butyrate, such as *Eubacterium*, *Flavonifractor* and *Subdoligranulum*, with cranberry juice supplementation^52,53^. In our study, *Clostridium*, *Clostridium* sensu stricto 1 were also increased, as well another known butyrate-producer, *Anaerobutyricum* (former *Eubacterium*) and *Shuttleworthia*^54–57^.

These changes were coherent with the SCFA quantified in both fecal and plasma samples. Indeed, it was observed that the fecal and plasmatic ratio of butyrate tended to increase in response to the cranberry extract, although the differences were not statistically significant. Interestingly, the increase in the butyrate ratio was concomitant to a decrease of the acetate ratio, despite the bloom of several acetate-producing bacteria such as *Terrisporobacter* (former *Clostridium*), *Intestinibacter* (former *Clostridium*), *Dorea* (former *Eubacterium*) and *Bifidobacterium*^58–60^. However, it has been previously demonstrated that *Bifidobacterium* promotes the growth and activity of butyrate-producing bacteria, leading to an increase production of butyrate in the gut. Species from *Bifidobacterium* can consume oligosaccharides to release acetate and lactate, and these intermediate metabolites can be converted into butyrate by specific bacteria, such as *Clostridium*, by a butyryl-CoA:acetate CoA transferase^61–63^. In the plasma, the increase in the ratio of butyrate was less pronounced compared to that in the feces, whereas the ratio of propionate showed an increase. However, no propionate-producing bacteria was stimulated by the cranberry extract supplementation in this study. Hence, this trend could be explained by the consumption of butyrate by colon epithelial cells, since this substrate is an important energy source for these cells^64^. Indeed, the decrease in the acetate ratio in the gut, coupled with the utilization of butyrate before it reaches systemic circulation, can indirectly lead to an increase in the ratio of propionate. As a result, the relative proportion of propionate in the bloodstream may appear higher due to these dynamic changes in acetate and butyrate metabolism.

An increase in butyrate production is recognized to be beneficial for human health. In fact, this key metabolite inhibits oxidative stress, inflammation and carcinogenesis in the gut, ameliorates the intestinal barrier and promotes satiety^64,65^. In addition, butyrate is reported to play a role in the prevention of type 2 diabetes. The microbiota of individuals with type 2 diabetes had a lower abundance of butyrate-producing bacteria; approaches to increase butyrate in the gut are considered promising to regulate glucose in those subjects^66^. Hence, cranberry extract is a potential prebiotic, as defined by the International Scientific Association for Probiotics and Prebiotics (ISAPP), by stimulating *Bifidobacterium*, a beneficial bacterial genus, and *Bifidobacterium* appears to use oligosaccharides from the cranberry extract to favor the production of butyrate, a beneficial metabolite for the gastrointestinal health of the host, by cross-feeding with butyrogenic bacteria^22^.

In addition to evaluating the overall impact of cranberry extract supplementation on the gut microbiota, we conducted enterotype-like clustering based on the changes observed in the fecal microbiota following supplementation. Interestingly, two enterotypes were characterized according to the differential modulation of the microbiota; one corresponded to the *Prevotella* and the other to the *Bacteroides* enterotypes previously reported^67^. In our study, 8 subjects belonged to the *Prevotella* enterotype (29%) and 20 subjects were included in the *Bacteroides* enterotype (71%), which is coherent with the reported prevalence of *Prevotella* in Western populations^68^. The cranberry extract supplementation increased the abundance of *Faecalibacterium* and *Agathobacter* in subjects from enterotype 1 (*Prevotella* enterotype), concomitant to a decrease of *Prevotella*. In contrast, participants belonging to enterotype 2 (*Bacteroides* enterotype) only benefited from the global effect, namely the bloom of *Bifidobacterium* at the expense of *Bacteroides*. In addition, *Phocaeicola* and *Bacteroides* were less reduced by the cranberry extract supplementation for subjects within enterotype 1 than those from enterotype 2. These enterotype-specific changes were coherent with the modulation of the SCFA. In enterotype 1, although not statistically significant, the supplementation led to a more pronounced increase in fecal and plasmatic butyrate ratios and a greater decrease in acetate ratios. This observation may be attributed to the bloom of butyrate-producing genera, such as *Faecalibacterium* and *Agathobacter*, within the gut microbiota of individuals belonging to enterotype 1^69^. In fact, it has been demonstrated that these two bacterial genera can produce butyrate by cross-feeding with *Bifidobacterium*^70,71^. In addition, the difference in plasmatic ratio of propionate is probably caused by the smaller decrease of propionate-producing bacteria, namely *Bacteroides* and *Phocaiecola* (formerly classified as *Bacteroides*)^72,73^. Thus, the inter-individual variability associated with the modulation of the fecal microbiota by the cranberry extract, was characterized into enterotypes. These specific enterotypes could explain the variations in the health outcomes resulting from the cranberry extract supplementation, since the gut microbiota of some individuals may produce more butyrate, a beneficial metabolite.

Interestingly, the effect of the initial composition of the gut microbiota on its differential response to oligosaccharides has previously been reported. In fact, the gut microbiota of elderly subjects was found to be modulated differently by arabinoxylan-oligosaccharides from wheat bran, depending on their enterotypes, specifically whether they belonged to the *Prevotella* or *Bacteroides* enterotype^74^. Interestingly, these molecules are able to induce a bifidogenic effect as observed with the cranberry extract^75,76^. However, in contrast to the cranberry extract, wheat bran oligosaccharides increased the abundance of *Prevotella* in participants belonging to the *Prevotella* enterotype^74^.

Surprisingly, *A. muciniphila* was not stimulated by the cranberry extract in our study. Previously, our group demonstrated that the abundance of this mucosal bacterial species, associated with antiobesity effect, was increased in mice fed with obesogenic diet supplemented with cranberry extract^23–25,77^. Nonetheless, the present study revealed that the impact on *A. muciniphila* was limited to individuals who already had this bacterium present in their microbiota prior to the administration of cranberry extract. This subset of samples accounted for approximately 46% of the total samples at V1 (Supplementary Fig. 12a). In most of these subjects, the abundance of this beneficial mucosal bacteria was predominantly increased, but, in certain individuals, it was decreased (Supplementary Fig. 12b). A possible explanation is the short duration of the supplementation in our study. *A. muciniphila* is a challenging bacterium in term of growth and may require longer supplementation to be stimulated. In fact, in our previous studies, *A. muciniphila* was stimulated after 8 to 9 weeks of cranberry extract supplementation in mice^23,24,77^. Also, fecal samples are not the best type of samples to assess the effect of the cranberry extract on *A. muciniphila*, since this bacteria is found in the mucus layer of the gut and the feces mostly represents the commensal bacteria found in the luminal environment^25,78^. The best sampling method to probe for this bacteria would be to carry a mucosal biopsy, but this technique is highly invasive, expensive and time-consuming^79^. A very good alternative is in vitro models, such as the Mucosal Simulator of the Human Intestinal Microbial Ecosystem (M-SHIME^®^), as we previously demonstrated the effect of ω-3 polyunsaturated fatty acids on *A. muciniphila* in the mucosal microbial niche of the transverse and descending colon using this dynamic fermentation system^80^. In addition, the absence of a cranberry extract effect on *A. muciniphila*, when this bacterium is not initially present, emphasizes the need to develop synbiotic combinations with a probiotic strain (such as *A. muciniphila*) and a potential prebiotic (like the cranberry extract)^81^.

In conclusion, this study is the first, to the best of our knowledge, to demonstrate the bifidogenic effect of a short-term supplementation with a cranberry extract rich in both (poly)phenols and oligosaccharides in a short-term human clinical trial. Further research should evaluate the long-term effect of this treatment, as well as the impact on health. Although not statistically significant, our study revealed interesting trends in short-chain fatty acid (SCFA) ratios, with an increase in the proportion of butyrate. This observation is particularly important as higher levels of butyrate have been linked to improved gastrointestinal health^64,65^. Also, it would be interesting to evaluate the effect of cranberry (poly)phenols and oligosaccharides separately, to validate the hypothesis of the duplibiotic effect of the cranberry extract and to use inulin as a control to compare the efficiency of the cranberry extract with a recognized prebiotic. Finally, larger cohorts are needed to validate the inter-individual variability associated with the modulation of the fecal microbiota by the cranberry extract, especially the bloom of *Faecalibacterium* for subjects belonging to the *Prevotella* enterotype.

## Methods

### Design of the clinical trial

To assess the impact of a cranberry extract on the fecal microbiota as a proxy for distal luminal colonic microbiota, 39 healthy subjects were enrolled from INAF’s volunteer database, representing the broader community of Quebec City. The selection criteria encompassed various factors, including the maintenance of a stable weight, not-smoking, no pregnancy and/or breastfeeding, following a consistent diet and physical activity routine, refrain from taking medication or experience stability in medication use for at least three months, and abstain from consuming antibiotics and/or probiotics for three months prior to the study. The characteristics of the enrolled subjects are displayed in Table 2.

The participants were instructed to refrain from consuming any food or beverage containing flavan-3-ols, as listed in Supplementary Table 5, for seven days before the intervention. Then, while adhering to these dietary restrictions, they took one cranberry extract capsule (Prebiocran^TM^) in the morning and one in the evening, providing 109.3 mg of (poly)phenols, as determined by ultra performance liquid chromatography coupled with ultraviolet detector and quadrupole – time of flight (UPLC-UV-QToF), and 125 mg of oligosaccharides per day for 4 days. The daily dose given to patients, comprising two capsules, is equivalent to the extract obtained from 60 g of fresh cranberries, following extraction and purification processes. Prior to and following the supplementation period, samples of feces and plasma were collected with a maximal delay of 2 h between the beginning/end of the treatment and the collection of biological samples. Fecal samples were collected in airtight containers by the participants, which included an anaerobic sachet (Fisher Scientific, Ottawa, Canada) to maintain oxygen-free conditions. The fecal samples were brought to the laboratory after a maximum of 2 h following the donation to prepare a fresh and anaerobic fecal slurry (20%, w/V). Each feces collected were suspended in anaerobic phosphate buffer (8.8 g/L K_2_HPO_4_, 6.8 g/L KH_2_PO_4_ and 1.0 g/L sodium thioglycolate) and homogenized using a Stomacher^TM^ lab blender to remove larger debris as previously described^82^. Both fecal and plasma samples were aliquoted and promptly stored at −80 °C until subsequent analysis.

This study was approved by the ethics committee for research involving human beings of Laval University under registration number: 2019-312 and informed consent was obtained from all the participants. The study was also registered at https://clinicaltrials.gov/ as NCT05931237.

### Characterization of the purified cranberry extract (Prebiocran™)

The purified cranberry extract named Prebiocran™ used in this study was provided by Symrise (Diana Food Canada Inc.). (Poly)phenolic composition was analyzed as previously reported by UPLC-UV-QToF^21,83^. Monosaccharide composition of oligosaccharides was determined by high-performance anion-exchange chromatography coupled with pulsed amperometric detection (HPAEC-PAD) following acid hydrolysis of the cranberry extract. The analysis was done on cranberry extract directly, rather than on the cranberry extract capsules, to avoid interference from the filling agent (hypromellose). Sample preparation was adapted from a previously published methodology^30^. Briefly, 10 mL of trifluoroacetic acid 2 M in water was added to 10 mg of cranberry extract. The resulting solution was vortexed for 30 s and then incubated at 120 °C for 2 h. Then, the solution was evaporated to dryness under a nitrogen stream, resuspended in 10 mL of isopropanol and re-evaporated to dryness under a nitrogen stream to eliminate residual acid. Before injection, the hydrolyzed cranberry extract was resuspended in 10 mM sodium hydroxide in water to a final concentration of 50 mg/L and passed through a Nylon 0.45 µm filter. The unhydrolyzed cranberry extract was also analyzed to confirm the absence of monosaccharides prior to the acid hydrolysis.

HPAEC-PAD analysis was performed with a Dionex^TM^ ICS-6000 (Thermo Scientific, Waltham, MA, USA). 10 µL of sample were injected onto a CarboPac PA20 (3 × 150 mm, 6.5 µm) (Thermo Scientific, Waltham, MA) protected with a guard column CarboPac PA20 (3 × 30 mm, 6.5 µm) (Thermo Scientific, Waltham, MA) heated to 30 °C. Tertiary gradient was performed with water (mobile phase A), 200 mM NaOH in water (mobile phase B) and 200 mM NaOH and 125 mM sodium acetate in water (mobile phase C) at a flow rate of 0.4 mL/min. The gradient started with 97.5% A and 2.5% B, then the proportion of mobile phase B was increased to 100% over 30 minutes. After the elution was done for 15 minutes with 100% C. Finally, the column was washed off for 20 minutes with 100% B and re-equilibrated for 24 minutes with initial conditions. Samples were kept at 8 °C in the autosampler compartment.

### DNA extraction

Fecal DNA was isolated from the pellets obtained by centrifuging 500 µL of fecal slurry using the Zymo Research kit (Quick-DNA^TM^ Fecal/Soil Microbe MiniPrep Kit) following the manufacturer’s protocol (Zymo Research, Irvine, CA). Enzymatic lysis of DNA was performed using lysozyme (20 mg) and mutanolysine (10 KU) (Sigma-Aldrich, Oakville, Canada). The resulting DNA extracts were eluted in 1X TE buffer (Tris and EDTA) and stored at −20 °C until sequencing. DNA quality was assessed by gel electrophoresis (1.2% w/v agarose) (Life Technologies, Madrid, Spain). DNA concentrations were determined using a Qubit (Thermo Fisher Scientific, Waltham, US). DNA samples were stored at −20 °C until preparation of the 16 S rRNA library.

### Fecal microbiota profiling by 16 S rRNA sequencing

The primer pairs F (5′-TCGTCGGCAGCGTCAGATGTGTATAAGAGACAGCCTACGGGNGGCWGCAG-3′) and R (5′-GTCTCGTGGGCTCGGAGATGTGTATAAGAGACAGGACTACHVGGGTATCTAATCC-3′) (341F-805R) were employed to amplify the V3-V4 hypervariable region of the 16 S rRNA gene. As suggested by the Qiaseq 16 S Region panel protocol (Qiagen, Hilden, Germany), the QIAseq 16 S/ITS 384-Index I kit (Qiagen) was used to prepare the amplicon library. To assess the quality and size of the 16 S metagenomic libraries, an Agilent High Sensitivity DNA Kit (Agilent, Palo Alto, US) was used in conjunction with a Bioanalyzer. The libraries were quantified using both a Quant-iT PicoGreen dsDNA Kit (Thermo Fisher Scientific, Waltham, MA, USA) and a Qubit (Thermo Fisher Scientific, Waltham, MA, USA). Subsequently, the PCR products were pooled and paired-end sequencing was performed using the MiSeq 600 cycles Reagent Kit V3 on an Illumina MiSeq System (Illumina, San Diego, USA).

The demultiplexed raw data files encompassing all the samples were imported into the R Studio 2022.12.0 environment using R version 4.1.3. The DADA2 R package (version 1.20.0) was employed to infer the amplicon sequence variants (ASV), following the recommended workflow^84^. Initially, sequence reads underwent filtering and trimming utilizing specific parameters: truncQ = 2, truncLen = c(250, 215) and maxEE = c(2,2). Filtered reads were then subjected to denoising using the DADA2 algorithm, which estimates sequencing errors. Chimeric sequences were removed and the ASV sequences were subsequently merged and classified using the SILVA database SSU Ref NR 99 release 138 with default parameters^85^. Unassigned taxa and singletons were excluded from the dataset. To account for variations in sampling depth, the data were rescaled to proportions for further analysis.

### qPCR assay

The qPCR procedure was conducted on the Applied biosystem 7500 instrument, employing primers targeting 16S total microbiota (Uni334F: 5’-ACTCCTACGGGAGGCAGCAGT-3’; Uni514R: 5’-ATTACCGCGGCTGCTGGC-3’), *Bifidobacterium* (F: 5’-TCGCGTCYGGTGTGAAAG-3’; R: 5’-CCACATCCAGCRTCCAC-3’) and *Bacteroides* (F: 5’-GGTGTCGGCTTAAGTGCCAT-3’; R: 5’-CGGAYGTAAGGGCCGTGC-3’)^86,87^. Each reaction was run in duplicate in a 96-well reaction plate sealed and maintained at a final volume of 15 µL. The qPCR reaction and thermal cycling amplification procedure followed was previously described^88^. Absolute quantification of gene copy numbers utilized standard curves obtained through droplet digital PCR (ddPCR) on the IBIS (Institute for Integrative Systems Biology, Laval University) sequencing platform, as described previously^89^.

### SCFA analysis in feces and plasma

Feces and plasma were extracted with methyl tert-butyl ether and analyzed by gas chromatography coupled with flame ionization detector (GC-FID) as previously published^82,90^. Acetate, propionate, butyrate, isobutyrate, valerate, isovalerate and hexanoate were quantified. However, since no significant difference, nor interesting trend were obtained with isobutyrate, valerate, isovalerate and hexanoate, only acetate, propionate and butyrate were reported. SCFA analysis was performed in duplicate. Results were averaged.

### Statistical analysis

Statistical analysis was conducted using R version 4.3.0 in the R Studio 2023.03.1 environment. Each measurement was taken from distinct samples. Shannon and Chao1 indexes were calculated with the package vegan (2.6-4). Using the same package, db-RDA was performed with Bray-Curtis distance and the statistical significance of the effect of the cranberry extract supplementation was assessed by permutational multivariate analysis of variance (PERMANOVA). The package DESeq2 (1.40.1) was used to perform differential analysis of normalized counts between conditions (effect of the cranberry extract supplementation or differences between enterotypes) and the Wald test adjusted for multiple comparisons using the Benjamini & Hochberg method was used to assess statistical significance. PCA was carried out using the package FactoMineR (2.8). Clustering was done by k-means algorithm using the package stats (4.3.0). All statistical comparison, except for DESeq analysis, was performed with the package rstatix (0.7.2). Shapiro-Wilk test was performed to assess normality. When data were normally distributed, paired-*t* test (two-sided) was performed, while Wilcoxon rank sum test (two-sided) was used for non-normal distributions. The paired version of these tests was used to assess the effect of the cranberry extract supplementation (V1 *vs* V2). Adjustment for multiple comparisons was carried out with Benjamini & Hochberg method. Finally, all graphics were generated with the packages ggplot2 (3.4.2), ggpubr (0.6.0) and ggrepel (0.9.3).

### Reporting summary

Further information on research design is available in the Nature Research Reporting Summary linked to this article.

### Supplementary information

Supplemental Material

Reporting Summary

## Data Availability

Raw 16S rRNA gene amplicon sequencing data were made publicly available online through the Sequence Read Archive (SRA) portal of NCBI under accession number PRJNA955174.

## Code availability

The code used for the bioinformatic and statistical analysis is available upon request to the authors.
