# Supplementary material for: Short term supplementation with cranberry extract modulates gut microbiota in human and displays a bifidogenic effect
Source: NPJ Biofilms Microbiomes. 2024 Mar 6;10:18. doi: 10.1038/s41522-024-00493-w (PMC10918075; doi:10.1038/s41522-024-00493-w)
Supplement: Supplementary file 1 — Supplemental Material [file 41522_2024_493_MOESM1_ESM.pdf]

Electronic supplementary material (ESI)

Short term supplementation with cranberry extract modulates gut microbiota in human and displays bifidogenic effect

Jacob Lessard-Lord[a,b,c], Charlène Roussel[a,b,d], Joseph Lupien-Meilleur[a,b,e], Pamela Généreux[a,e], Véronique Richard[a], Valérie Guay[a,b], Denis Roy[a,e] and Yves Desjardins[a,b,c*]

Affiliations

a) Institute of Nutrition and Functional Foods (INAF), Faculty of Agriculture and Food Sciences, Laval University, Québec, QC, Canada
b) Nutrition, Health and Society Centre (NUTRISS), INAF, Laval University, Québec, QC, Canada
c) Department of Plant Science, Faculty of Agriculture and Food Sciences, Laval University, Québec, QC, Canada
d) Canada Excellence Research Chair on the Microbiome-Endocannabinoidome Axis in Metabolic Health, Laval University, Québec, QC, Canada
e) Department of Food Science, Faculty of Agriculture and Food Sciences, Laval University, Québec, QC, Canada

*Corresponding author. *E-mail address:* yves.desjardins@fsaa.ulaval.ca

Supplementary Table 1. Detailed characterization of the (poly)phenols provided by the cranberry extract.

Class	Compound	Daily dose (mg)		
**Flavan-3-ols**	Catechin	0,1293	±	0,0017
	Epicatechin	1,865	±	0,017
	Procyanidin A2	4,46	±	0,04
	Procyanidin B2	*Non detected*		
	**Flavan-3-ols total[a]**	**82,3**	**±**	**0,2**
**Phenolic acids**	Coumaric acid	2,315	±	0,017
	Coumaroyl-hexoside - 1	0,415	±	0,004
	Coumaroyl-hexoside - 2	0,1760	±	0,0008
	Coumaroyl-monotropein	0,337	±	0,003
	Coumaroyl-dihydromonotropein	0,716	±	0,005
	Neochlorogenic acid	*Non detected*		
	Chlorogenic acid	1,795	±	0,015
	Cryptochlorogenic acid	*Non detected*		
	Caffeoyl-hexoside	0,399	±	0,004
	Sinapoyl-hexoside	0,403	±	0,003
	Vanilloyl-hexoside	0,0676	±	0,0008
	Feruloyl-hexoside	0,254	±	0,002
	3,4-dihydroxybenzoic acid	*Non detected*		
	Dihydroxybenzoic acid - 1	0,1285	±	0,0014
	Dihydroxybenzoic acid - 2	0,0621	±	0,0007
	Dihydroxybenzoyl-hexoside	0,0451	±	0,0006
	Hydroxybenzoyl-hexosyl-hexoside	0,1388	±	0,0013
	**Phenolic acids and derivatives total**	**7,25**	**±**	**0,05**
**Flavonols**	Quercetin	4,75	±	0,06
	Quercetin-3-galactoside	1,897	±	0,016
	Quercetin-3-rhamnoside	0,996	±	0,009
	Quercetin-pentoside - 1	0,662	±	0,005
	Quercetin-pentoside - 2	0,421	±	0,003
	Quercetin-pentoside - 3	0,394	±	0,003
	Quercetin-deoxyhexoside	0,1125	±	0,0010
	Quercetin-hydroxybenzoyl-hexoside	0,385	±	0,004
	Isorhamnetin	0,329	±	0,003
	Myricetin	2,18	±	0,02
	Myricetin-pentoside 1	0,1036	±	0,0012
	Myricetin-pentoside - 2	0,171	±	0,003
	Myricetin-pentoside - 3	0,1449	±	0,0007
	Myricetin-hexoside	1,137	±	0,008
	Myricetin methyl	0,220	±	0,002
	Syringetin-hexoside	0,285	±	0,002
	**Flavonols total**	**14,18**	**±**	**0,12**
**Anthocyanins**	Cyanidin 3-galactoside	1,06	±	0,03
	Cyanidin 3-glucoside	0,106	±	0,002
	Cyanidin 3-arabinoside	1,70	±	0,04
	Cyanidin-3-xyloside	*Non detected*		
	Peonidin 3-galactoside	1,43	±	0,03
	Peonidin 3-glucoside	0,052	±	0,004
	Peonidin 3-arabinoside	1,16	±	0,02
	**Anthocyanins total**	**5,50**	**±**	**0,13**

[a] Determined by phloroglucinolysis. Flavan-3-ols in cranberry extract had a mean degree of polymerization of 5 and were composed of 33% of procyanidin A2 units.

Supplementary Table 2. Statistics obtained from DESeq2 analysis investigating the genera modulated by the cranberry extract.

Genus	log_2FoldChange	Adjusted p-value	Mean normalized raw counts	
			V1	V2
Bacteroides	-1.52	3.02E-09	2667.9	1121.5
Odoribacter	-1.55	1.18E-06	51.1	19.4
Sutterella	-1.96	1.70E-05	174.7	31.9
Parabacteroides	-1.19	2.79E-05	246.5	137.7
Parasutterella	-1.67	1.63E-04	90.6	56.0
Lachnospiraceae NK4A136 group	-1.14	2.04E-04	665.1	342.5
Barnesiella	-1.22	3.84E-04	176.0	68.6
Lachnospira	-1.52	3.84E-04	313.3	101.2
Terrisporobacter	1.73	3.84E-04	30.5	142.5
Bifidobacterium	1.16	3.93E-04	410.1	915.3
UCG-003	-1.42	7.21E-04	84.2	25.6
Lachnospiraceae UCG-004	-1.57	1.14E-03	62.4	28.3
Butyricimonas	-1.36	3.58E-03	8.0	2.2
Clostridium	1.23	4.73E-03	99.2	178.0
Alistipes	-0.66	8.86E-03	419.9	318.6
Oscillospira	-1.26	1.09E-02	16.0	1.9
Prevotella_9	-1.18	1.52E-02	1172.0	350.9
Intestinibacter	1.06	2.74E-02	104.8	176.2
[Eubacterium] eligens group	-0.96	2.93E-02	276.3	138.0
Paraprevotella	-1.11	2.93E-02	34.6	17.9
Clostridium sensu stricto 1	1.24	3.65E-02	53.8	235.7
Shuttleworthia	1.08	3.79E-02	3.3	7.1
Anaerobutyricum	0.44	3.90E-02	561.0	715.3
Dorea	0.41	4.02E-02	750.3	931.8

Supplementary Table 3. Statistics obtained from DESeq2 analysis investigating the species modulated by the cranberry extract.

ASV	Species	log_2FoldChange	Adjusted p-value	Mean normalized raw counts	
				V1	V2
ASV_24	*Bacteroides vulgatus*	-1.95	2.05E-06	385.9	118.7
ASV_243	*Odoribacter splanchnicus*	-1.98	7.64E-06	30.6	11.0
ASV_29	*Bacteroides uniformis*	-1.54	1.71E-05	343.2	139.8
ASV_204	*Bacteroides thetaiotaomicron*	-2.03	1.27E-04	42.9	7.9
ASV_141	*Bacteroides xylanisolvens*	-2.10	1.80E-04	56.9	12.1
ASV_96	*Terrisporobacter spp.*	1.64	2.12E-03	30.5	142.5
ASV_46	*Lachnospira pectinoschiza*	-1.62	2.31E-03	240.1	73.9
ASV_118	*Bacteroides vulgatus*	-1.27	4.01E-03	111.4	31.9
ASV_36	*Bacteroides uniformis*	-1.63	4.55E-03	204.8	201.9
ASV_190	*Lachnospiraceae UCG-004 spp.*	-1.61	4.61E-03	43.8	16.2
ASV_657	*UCG-003 spp.*	-1.51	7.96E-03	6.9	1.8
ASV_65	*Lachnospiraceae NK4A136 group spp.*	-1.30	9.68E-03	157.0	72.7
ASV_249	*Lachnospiraceae NK4A136 group spp.*	-1.49	9.72E-03	28.1	11.8
ASV_245	*Parasutterella excrementihominis*	-1.47	1.06E-02	34.8	13.7
ASV_53	*Clostridium disporicum*	1.13	1.41E-02	99.2	178.0
ASV_51	*[Eubacterium] eligens group spp.*	-1.22	1.43E-02	185.8	93.0
ASV_293	*Odoribacter splanchnicus*	-1.13	1.84E-02	20.5	8.3
ASV_62	*Lachnospiraceae NK4A136 group spp.*	-1.24	1.98E-02	170.7	85.4
ASV_270	*Parasutterella excrementihominis*	-1.25	2.48E-02	31.2	11.4
ASV_747	*Oscillospira spp.*	-1.16	2.48E-02	5.8	1.4
ASV_287	*Alistipes shahii*	-1.26	2.69E-02	21.9	7.6
ASV_64	*Clostridium sensu stricto 1 spp.*	1.35	2.69E-02	34.9	206.0
ASV_178	*Lachnospira pectinoschiza*	-1.21	2.80E-02	41.1	14.2
ASV_17	*Bifidobacterium adolescentis*	0.86	3.25E-02	258.5	589.9
ASV_170	*Bacteroides caccae*	-1.19	3.79E-02	43.4	36.4
ASV_55	*Bifidobacterium longum*	1.02	3.79E-02	67.3	153.2
ASV_384	*UCG-003 spp.*	-1.24	4.02E-02	18.6	4.0
ASV_52	*Intestinibacter bartlettii*	1.03	4.02E-02	104.8	176.2
ASV_35	*Alistipes putredinis*	-0.64	4.30E-02	191.1	132.8

Supplementary Table 4. Statistics obtained from DESeq2 analysis investigating the genera differentiating enterotype 1 and 2 at V1.

Genus	log_2FoldChange	Adjusted p-value	Mean normalized raw counts	
			Enterotype 1	Enterotype 2
Holdemanella	-7.78	1.51E-14	427.3	97.1
Alloprevotella	-5.15	2.70E-07	93.4	108.6
Ligilactobacillus	-4.30	5.32E-06	227.8	28.5
UBA1819	3.40	3.02E-05	2.6	21.7
Catenibacterium	-3.84	5.55E-05	248.8	81.9
Megasphaera	-3.43	9.37E-05	44.6	5.7
Turicibacter	3.87	9.37E-05	13.4	104.8
Prevotella_9	-5.35	3.77E-04	2223.2	176.8
Intestinibacter	3.67	9.67E-04	26.8	186.0
Rikenellaceae RC9 gut group	-2.91	9.67E-04	17.8	0.7
Tyzzerella	4.40	9.67E-04	0.0	39.7
Prevotella_7	-2.80	1.45E-03	8.4	0.0
Parasutterella	2.85	5.37E-03	9.9	98.7
Fournierella	2.95	1.82E-02	0.0	4.7
Eisenbergiella	2.58	2.24E-02	4.4	11.2
Gordonibacter	2.28	2.92E-02	0.0	3.6
Alistipes	1.21	3.80E-02	171.4	448.4
Bacteroides	1.04	3.80E-02	1070.4	2224.5
Erysipelatoclostridium	2.38	3.80E-02	9.0	74.5
Fusicatenibacter	1.51	3.80E-02	679.5	1541.2
Prevotellaceae NK3B31 group	-1.78	4.26E-02	6.6	130.3
Acidaminococcus	2.52	4.85E-02	0.0	14.1
Granulicatella	-1.69	4.85E-02	2.8	1.0

Supplementary Table 5. Food and beverage containing flavan-3-ols participants were asked to not consume during the study.

Category	Item
**Fruits**	**All the fruits except :**
	Citrus
	Pineapple
	Watermelon
**Vegetables**	Indian squash
	Rhubarb
**Legumes**	All beans
	Lentil
	Cowpea
	Carob
**Cereals**	Soy (including tofu, etc.)
	Barley
	Buckwheat
	Sorghum
	Millet
	Rice
**Beverages**	Tea
	Coffee
	Juice of the fruits to avoid
	Wine
	Beer
**Others**	Chocolate
	Nuts and peanut
	Avocado
	Cinnamon
	Curry

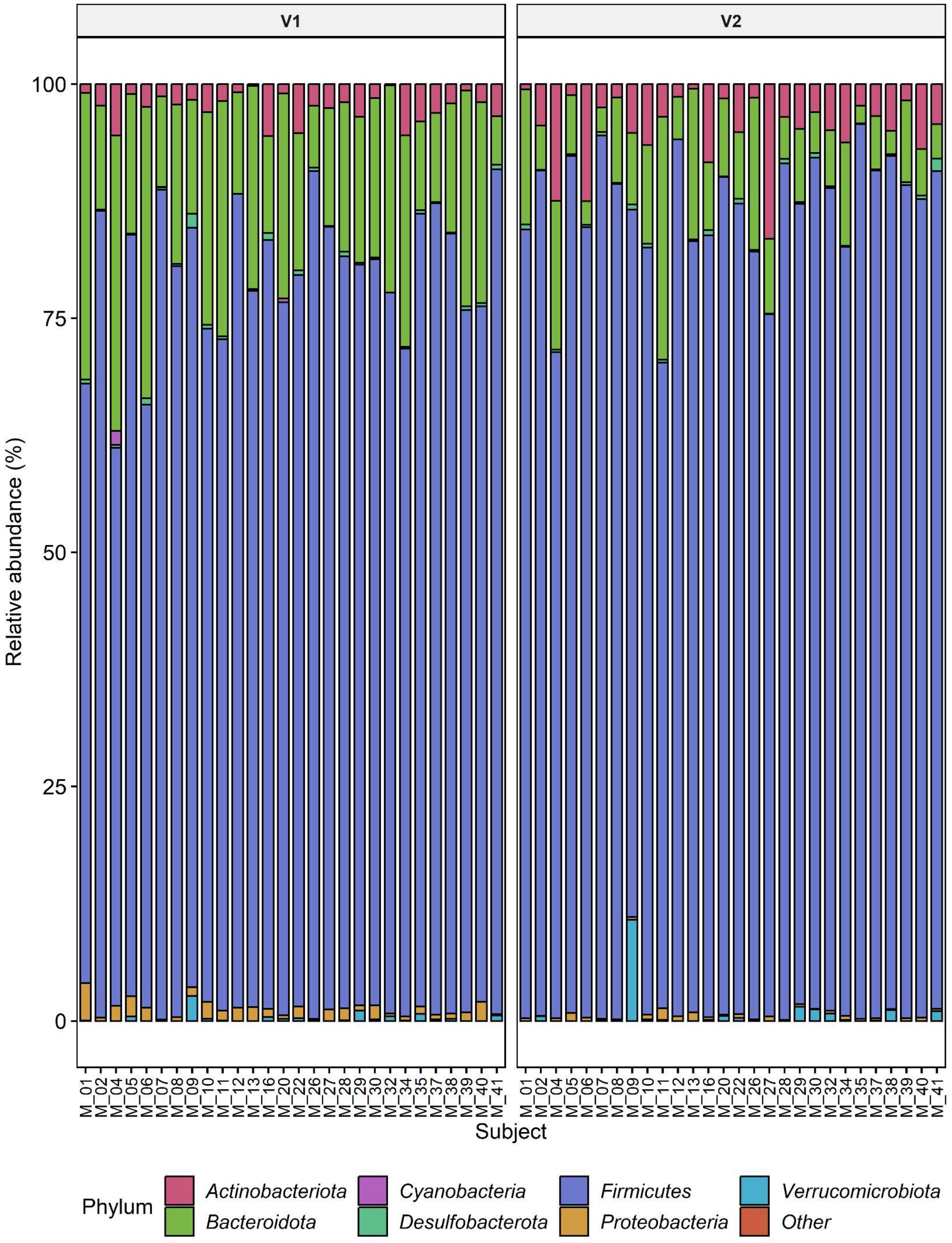

Supplementary Figure 1. Microbial composition of each subject before (V1) and after (V2) the cranberry extract supplementation at the phylum level.

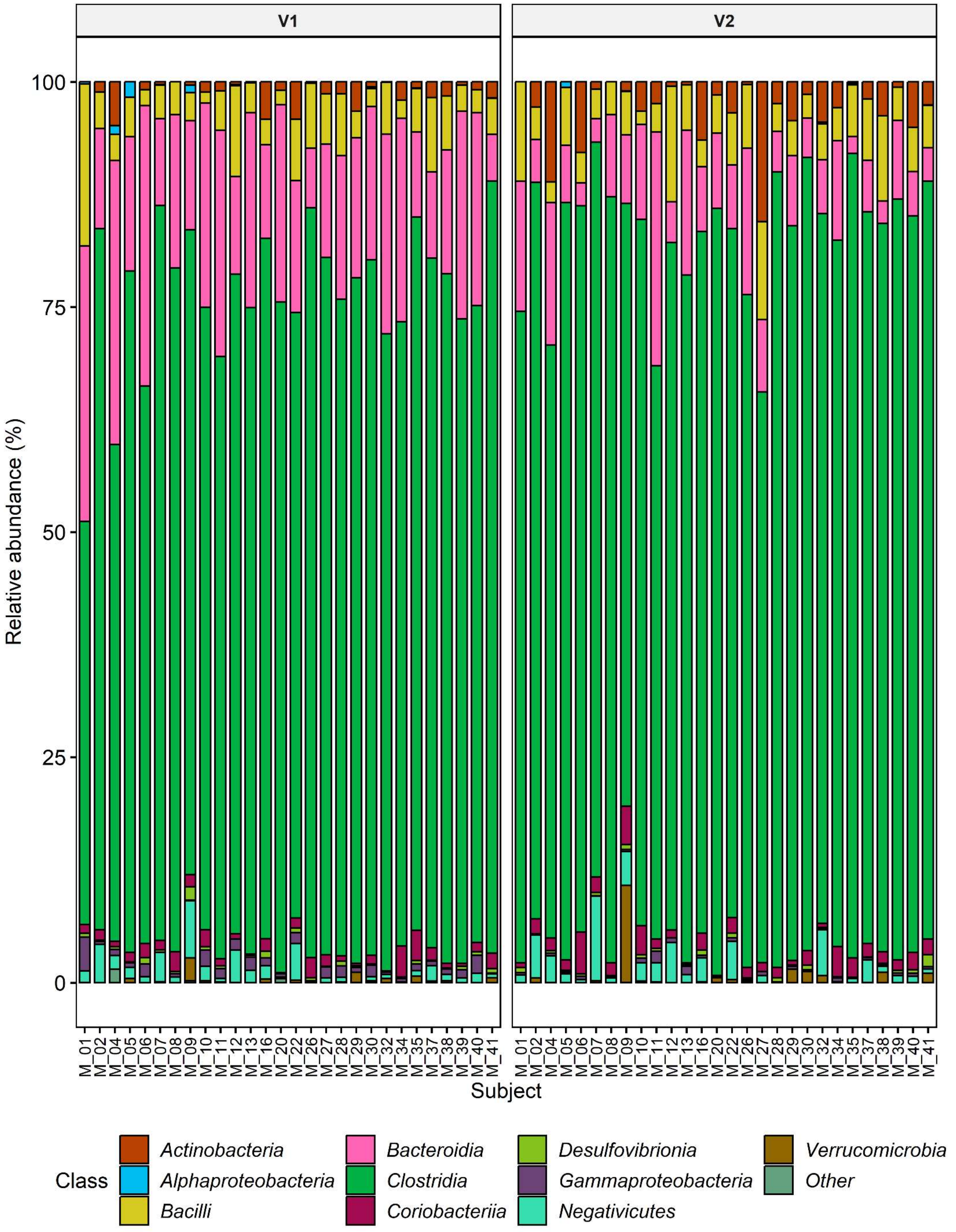

Supplementary Figure 2. Microbial composition of each subject before (V1) and after (V2) the cranberry extract supplementation at the class level.

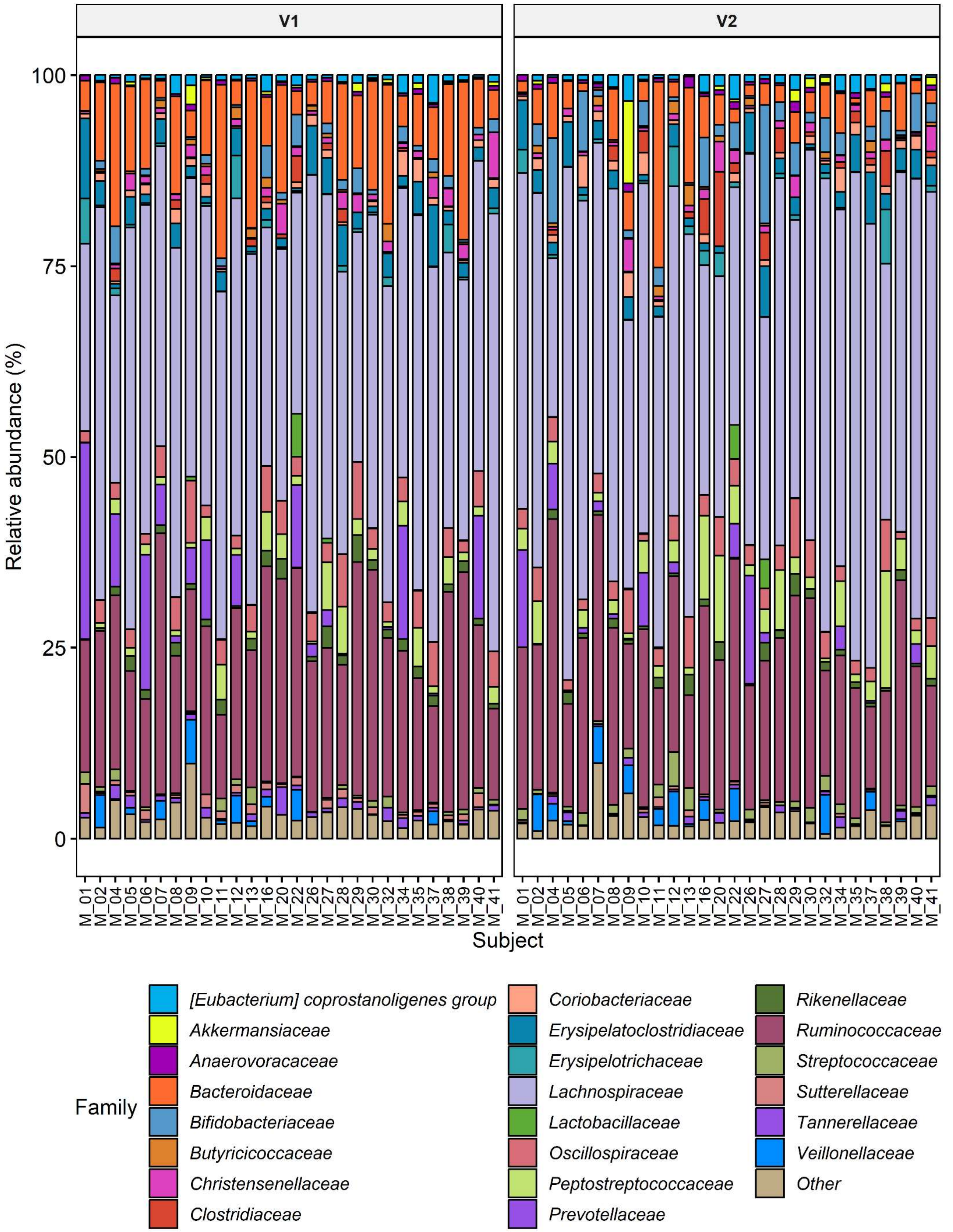

Supplementary Figure 3. Microbial composition of the 22 most abundant families for each subject before (V1) and after (V2) the cranberry extract supplementation.

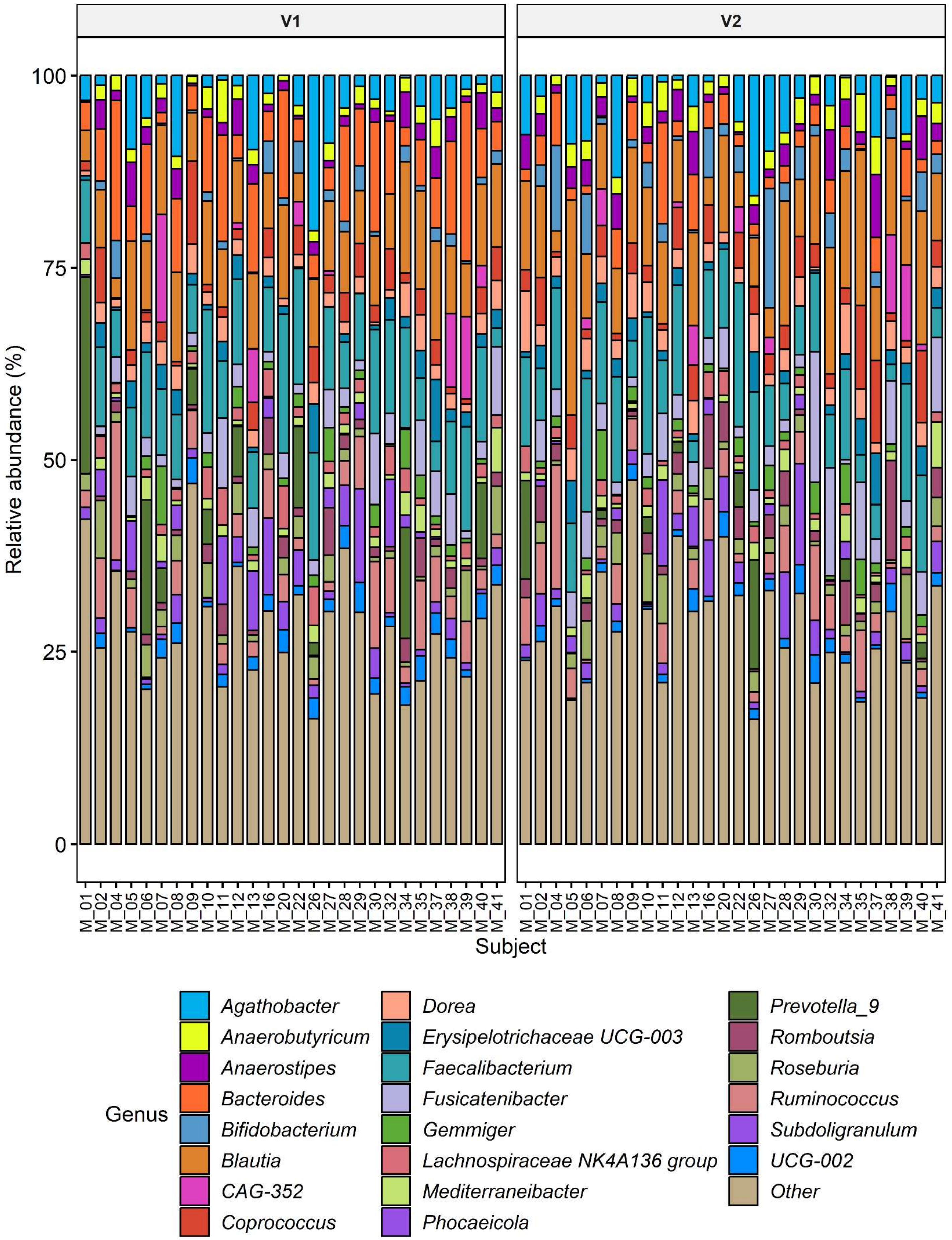

Supplementary Figure 4. Microbial composition of the 22 most abundant genera for each subject before (V1) and after (V2) the cranberry extract supplementation.

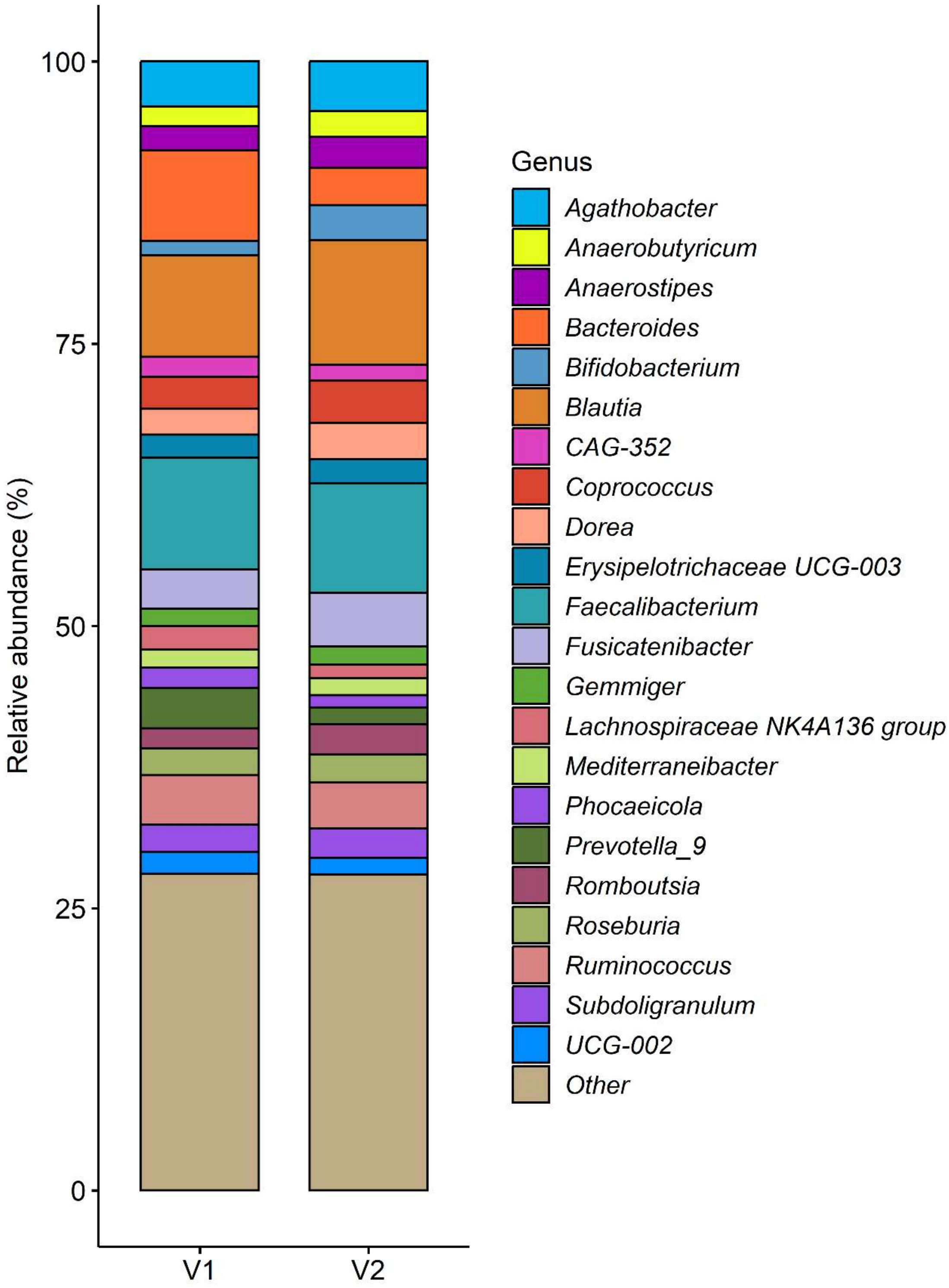

Supplementary Figure 5. Mean microbial composition of all subjects before (V1) and after (V2) the cranberry extract supplementation at the genus level. The 22 most abundant genera are indicated in the plot. Results are expressed as the mean of the 28 subjects.

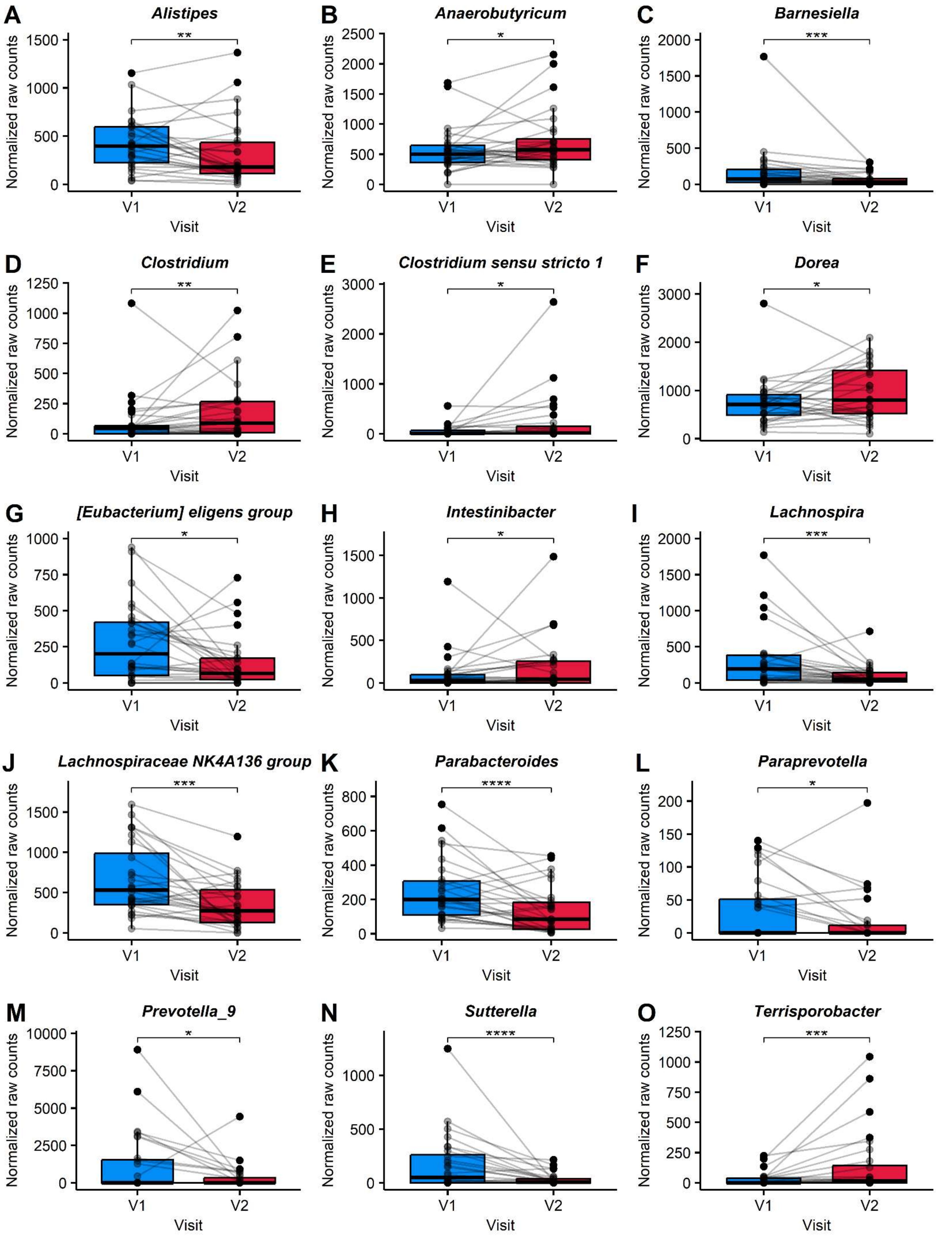

Supplementary Figure 6. Bacterial genera significantly modulated by the cranberry extract supplementation as assessed by DESeq2 analysis.

Statistical significance was assessed with Wald test adjusted for multiple comparisons using the Benjamini & Hochberg method. The results were represented with asterisk (* : $p \leq 0.05$, ** : $p \leq 0.01$, *** : $p \leq 0.001$, **** : $p \leq 0.0001$).

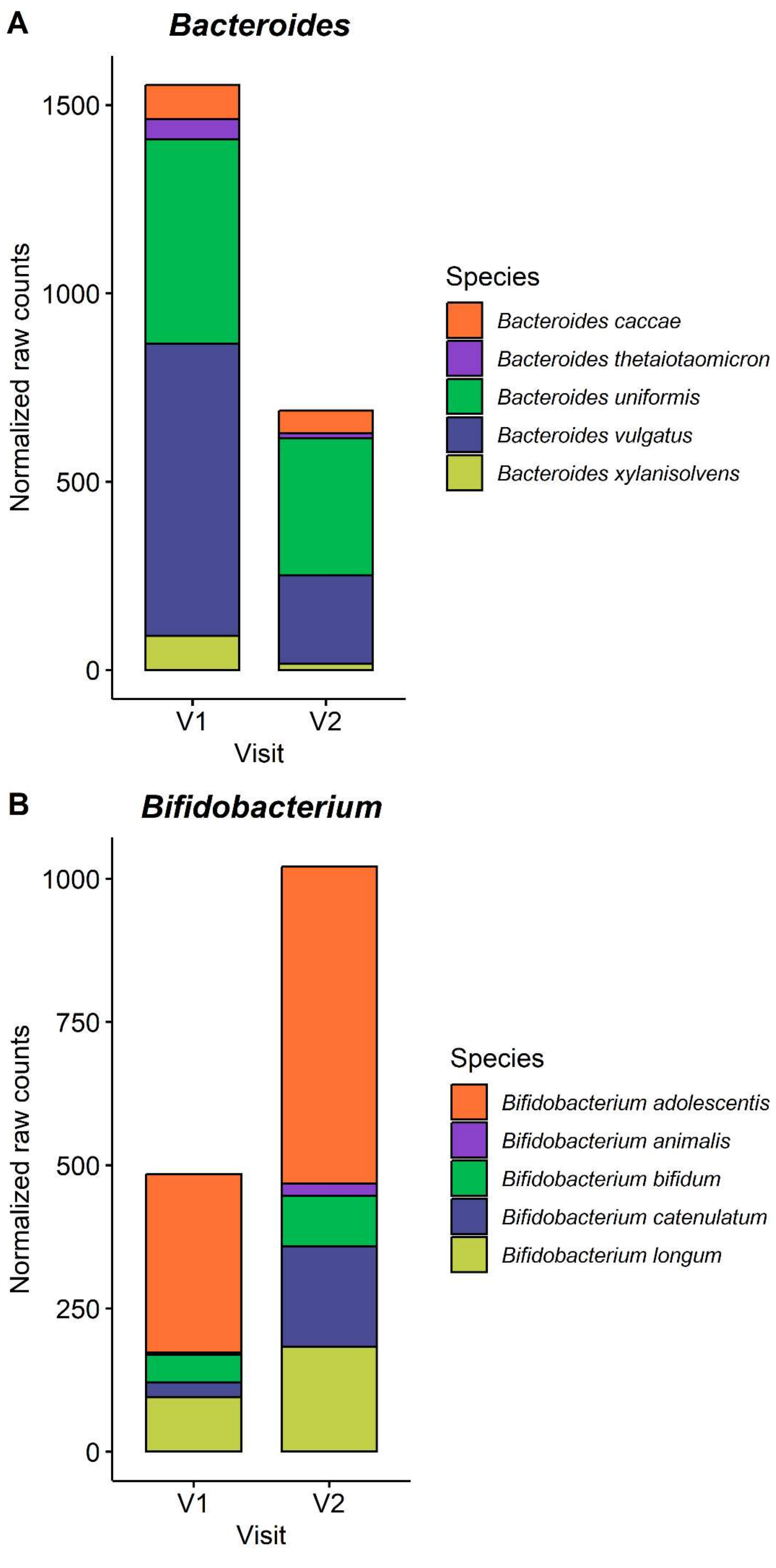

Supplementary Figure 7. Abundance of specific species of *Bacteroides* (A) and *Bifidobacterium* (B) assigned with RDP classifier.

Results are expressed as the mean of the 28 subjects. ASV belonging to the same species were summed. For *Bacteroides* (A), only significant species (p-value ≤ 0.05, as assessed by DESeq2 analys) were represented.

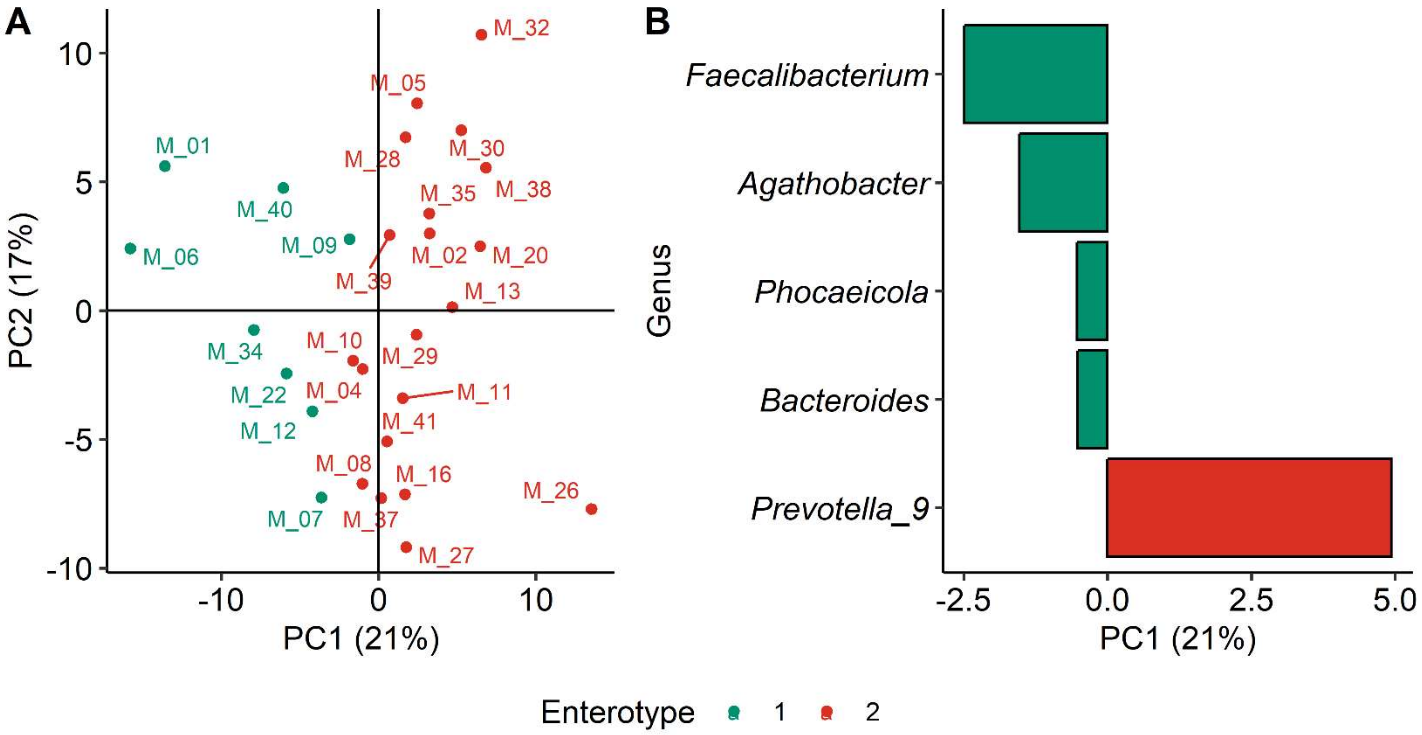

Supplementary Figure 8. Stratification of the subjects into enterotypes based on the modulation of their fecal microbiota by cranberry extract supplementation. PCA scores (A) and loadings (B) plot at the genus level are presented based on the differential relative abundance (V2 – V1) of taxa. Data were not scaled prior to PCA. % of the total variability explained by each PC is reported in axis titles.

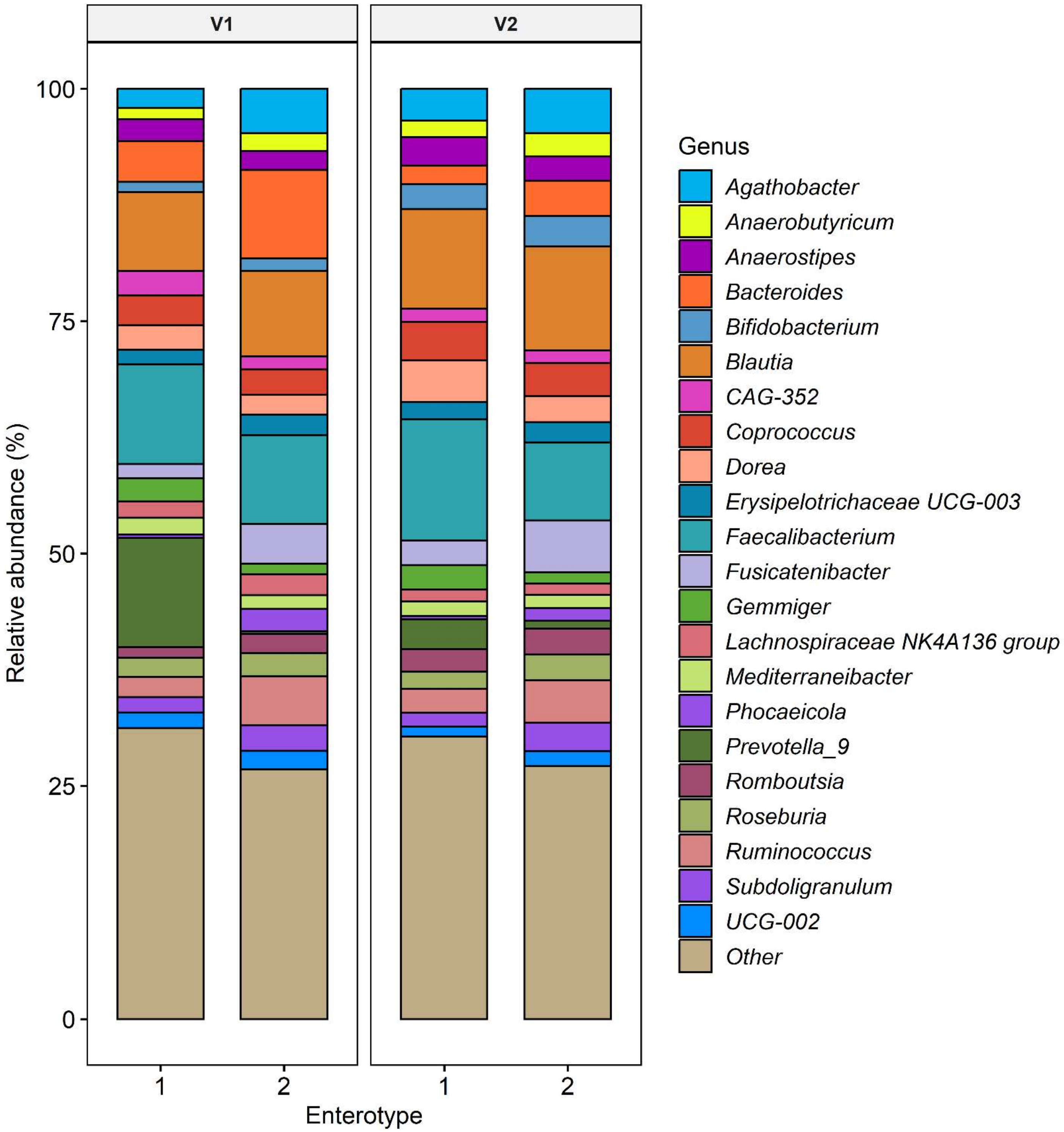

Supplementary Figure 9. Microbial composition of each enterotype before (V1) and after (V2) the cranberry extract supplementation at the genus level. The 22 most abundant genera are indicated in the plot. Results are expressed as the mean of the 8 subjects within enterotype 1 and the 20 subjects belonging to enterotype 2.

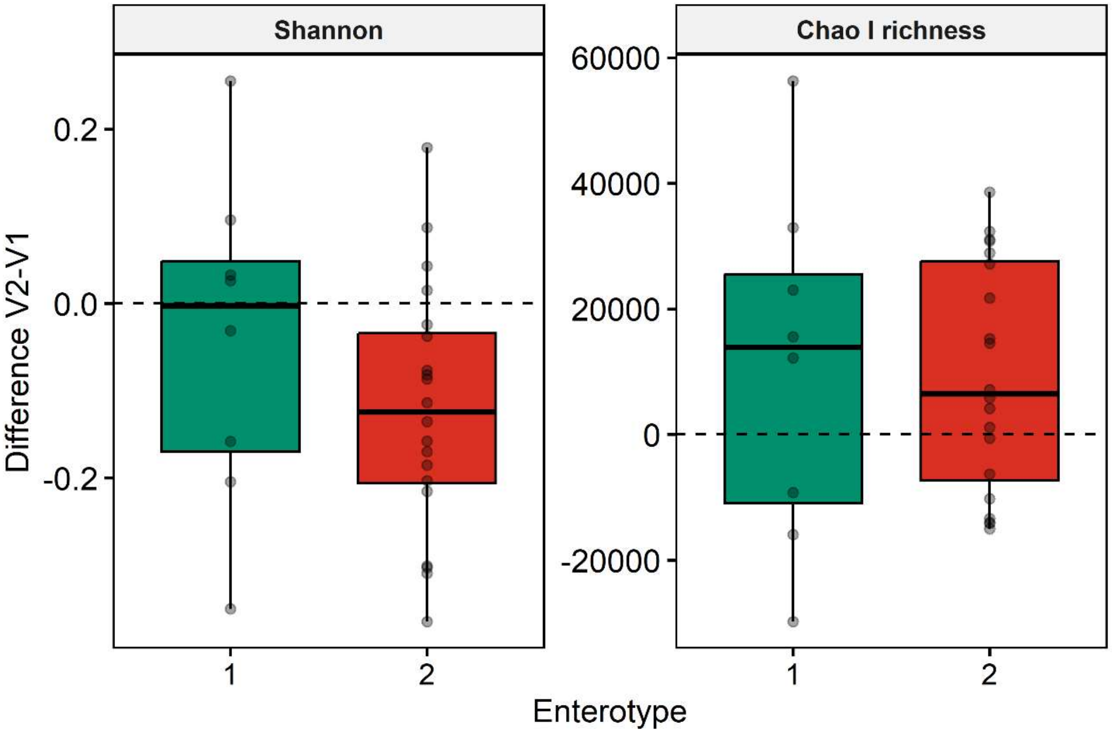

Supplementary Figure 10. Impact of the cranberry extract supplementation on α-diversity and richness depending on the enterotypes. Statistical significance was assessed with Wilcoxon test for multiple comparisons using the Benjamini & Hochberg method. No significant difference was found between enterotypes.

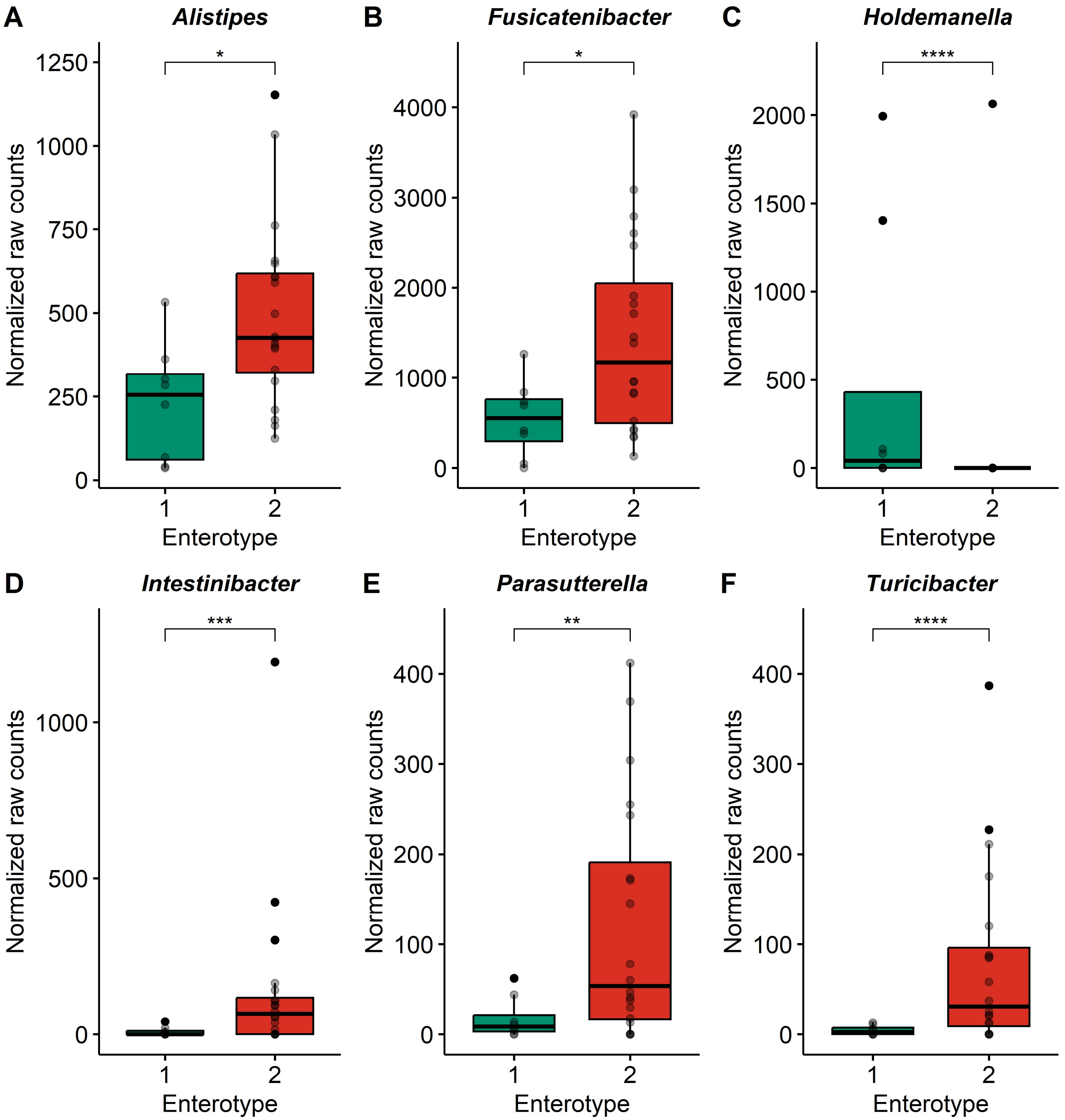

Supplementary Figure 11. Bacterial genera significantly differentiating enterotypes before the cranberry extract supplementation (V1) assessed by DESeq2 analysis. Statistical significance was assessed with Wald test adjusted for multiple comparisons using the Benjamini & Hochberg method. The results were represented with asterisk (* : $p \leq 0.05$, ** : $p \leq 0.01$, *** : $p \leq 0.001$, **** : $p \leq 0.0001$).

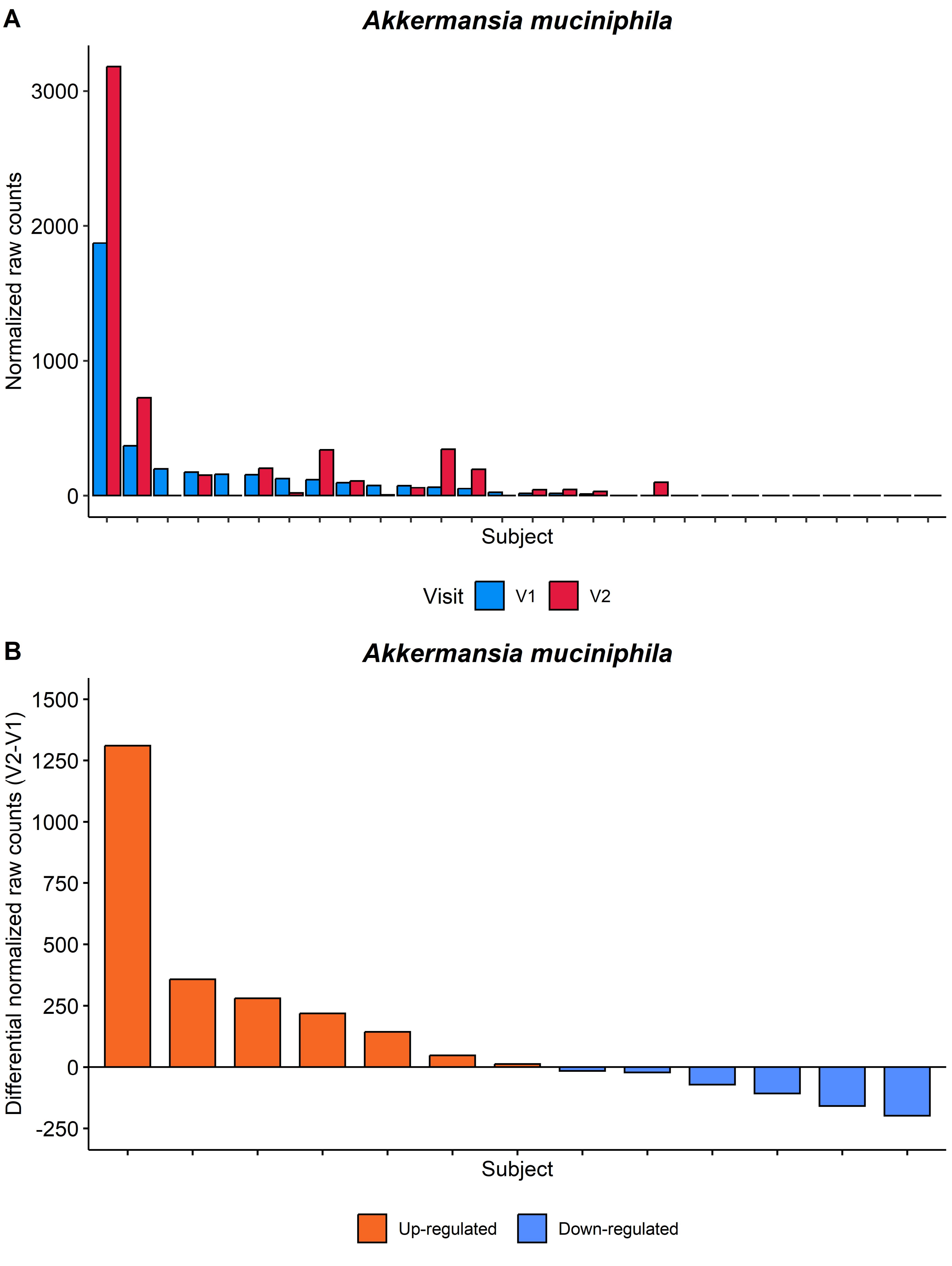

Supplementary Figure 12. Impact of the cranberry extract on *Akkermansia muciniphila*.

(A) Abundance of *A. muciniphila* before (V1) and after (V2) the cranberry extract supplementation in all participants. (B) Differential abundance of *A. muciniphila* (V2 -V1) in subjects with *A. muciniphila* in their initial fecal sample (V1).
