# Supplementary material for: Short term supplementation with cranberry extract modulates gut microbiota in human and displays a bifidogenic effect
Source: NPJ Biofilms Microbiomes. 2024 Mar 6;10:18. doi: 10.1038/s41522-024-00493-w (PMC10918075; doi:10.1038/s41522-024-00493-w)
Supplement: Supplementary file 2 — Reporting Summary [file 41522_2024_493_MOESM2_ESM.pdf]

nature portfolio

Corresponding author(s): Yves Desjardins

Last updated by author(s): Jan 16, 2024

Reporting Summary

Nature Portfolio wishes to improve the reproducibility of the work that we publish. This form provides structure for consistency and transparency in reporting. For further information on Nature Portfolio policies, see our Editorial Policies and the Editorial Policy Checklist.

Statistics

For all statistical analyses, confirm that the following items are present in the figure legend, table legend, main text, or Methods section.

n/a	Confirmed	
☐	☒	The exact sample size (*n*) for each experimental group/condition, given as a discrete number and unit of measurement
☐	☒	A statement on whether measurements were taken from distinct samples or whether the same sample was measured repeatedly
☐	☒	The statistical test(s) used AND whether they are one- or two-sided<br>*Only common tests should be described solely by name; describe more complex techniques in the Methods section.*
☒	☐	A description of all covariates tested
☐	☒	A description of any assumptions or corrections, such as tests of normality and adjustment for multiple comparisons
☒	☐	A full description of the statistical parameters including central tendency (e.g. means) or other basic estimates (e.g. regression coefficient) AND variation (e.g. standard deviation) or associated estimates of uncertainty (e.g. confidence intervals)
☐	☒	For null hypothesis testing, the test statistic (e.g. F, t, r) with confidence intervals, effect sizes, degrees of freedom and P value noted<br>*Give P values as exact values whenever suitable.*
☒	☐	For Bayesian analysis, information on the choice of priors and Markov chain Monte Carlo settings
☒	☐	For hierarchical and complex designs, identification of the appropriate level for tests and full reporting of outcomes
☒	☐	Estimates of effect sizes (e.g. Cohen's *d*, Pearson's *r*), indicating how they were calculated

Our web collection on statistics for biologists contains articles on many of the points above.

Software and code

Policy information about availability of computer code

Data collection	No software were used.
Data analysis	Statistical analysis was conducted using R version 4.3.0 in the R Studio 2023.03.1 environment.

For manuscripts utilizing custom algorithms or software that are central to the research but not yet described in published literature, software must be made available to editors and reviewers. We strongly encourage code deposition in a community repository (e.g. GitHub). See the Nature Portfolio guidelines for submitting code & software for further information.

Data

Policy information about availability of data

All manuscripts must include a data availability statement. This statement should provide the following information, where applicable:

- Accession codes, unique identifiers, or web links for publicly available datasets
- A description of any restrictions on data availability
- For clinical datasets or third party data, please ensure that the statement adheres to our policy

Raw 16S rRNA gene amplicon sequencing data were made publicly available online through the Sequence Read Archive (SRA) portal of NCBI under accession number PRJNA955174.

Research involving human participants, their data, or biological material

Policy information about studies with human participants or human data. See also policy information about sex, gender (identity/presentation), and sexual orientation and race, ethnicity and racism.

Reporting on sex and gender	We did not carry sex-stratified analysis due to low statistical power (n = 39 total), but only 28 provided fecal samples before and after the supplementation.
Reporting on race, ethnicity, or other socially relevant groupings	We did not collect information about the ethnicity of our participants.
Population characteristics	In our study, the mean age of the participants was 36 (SD = 11). Out of the 39 participants, 24 were female and 15 were male. The average weight was 67 kg (SD = 13), average BMI was 23 kg/m2 (SD = 4) and average waist circumference was 82 cm (SD = 13).
Recruitment	39 healthy subjects were enrolled from INAF's volunteer database, representing the broader community of Quebec City.
Ethics oversight	Ethics committee for research involving human beings of Laval University

Note that full information on the approval of the study protocol must also be provided in the manuscript.

Field-specific reporting

Please select the one below that is the best fit for your research. If you are not sure, read the appropriate sections before making your selection.

☒ Life sciences ☐ Behavioural & social sciences ☐ Ecological, evolutionary & environmental sciences

For a reference copy of the document with all sections, see nature.com/documents/nr-reporting-summary-flat.pdf

Life sciences study design

All studies must disclose on these points even when the disclosure is negative.

Sample size	We did not calculate sample size before the study. Sample size was chose based on the budget available at the time.
Data exclusions	No data were excluded.
Replication	The clinical trial was not repeated, but the bifidogenic effect was observed across the participants.
Randomization	There was no randomization, since every participant was his own control.
Blinding	Blinding was not possible, since all participants did the control period first and after the supplementation.

Reporting for specific materials, systems and methods

We require information from authors about some types of materials, experimental systems and methods used in many studies. Here, indicate whether each material, system or method listed is relevant to your study. If you are not sure if a list item applies to your research, read the appropriate section before selecting a response.

Materials & experimental systems

n/a	Involved in the study	
☒	☐	Antibodies
☒	☐	Eukaryotic cell lines
☒	☐	Palaeontology and archaeology
☒	☐	Animals and other organisms
☒	☐	Clinical data
☒	☐	Dual use research of concern
☒	☐	Plants

Methods

n/a	Involved in the study	
☒	☐	ChIP-seq
☒	☐	Flow cytometry
☒	☐	MRI-based neuroimaging

Plants

Seed stocks	NA
Novel plant genotypes	NA
Authentication	NA
